# The RNA‐binding protein MEX3A is a prognostic factor and regulator of resistance to gemcitabine in pancreatic ductal adenocarcinoma

**DOI:** 10.1002/1878-0261.12847

**Published:** 2020-11-24

**Authors:** Valentina Panzeri, Isabella Manni, Alessia Capone, Chiara Naro, Andrea Sacconi, Silvia Di Agostino, Luisa de Latouliere, Andrea Montori, Emanuela Pilozzi, Giulia Piaggio, Gabriele Capurso, Claudio Sette

**Affiliations:** ^1^ Department of Science Medical/Chirurgic and Translational Medicine University of Rome “Sapienza” Italy; ^2^ Department of Neuroscience Section of Human Anatomy Catholic University of the Sacred Heart Rome Italy; ^3^ UOSD SAFU Department of Research, Diagnosis and Innovative Technologies IRCCS Regina Elena National Cancer Institute Rome Italy; ^4^ Fondazione Santa Lucia IRCCS Rome Italy; ^5^ IRCCS Fondazione Policlinico Agostino Gemelli Rome Italy; ^6^ Clinical Trial Center, Biostatistics and Bioinformatics Unit IRCCS Regina Elena National Cancer Institute Rome Italy; ^7^ Oncogenomic and Epigenetic Unit Department of Diagnostic Research and Technological Innovation IRCCS Regina Elena National Cancer Institute Rome Italy; ^8^ Department of Clinical and Molecular Medicine UOC Anatomia Patologica Sant' Andrea Hospital Sapienza University of Rome Italy; ^9^ PancreatoBiliary Endoscopy and EUS Division Pancreas Translational and Clinical Research Center San Raffaele Scientific Institute IRCCS Milan Italy

**Keywords:** cell cycle, chemoresistance, PDAC, RNA‐binding proteins, RNA metabolism

## Abstract

Pancreatic ductal adenocarcinoma (PDAC) is a highly aggressive cancer. Most patients present with advanced disease at diagnosis, which only permits palliative chemotherapeutic treatments. RNA dysregulation is a hallmark of most human cancers, including PDAC. To test the impact of RNA processing dysregulation on PDAC pathology, we performed a bioinformatics analysis to identify RNA‐binding proteins (RBPs) associated with prognosis. Among the 12 RBPs associated with progression‐free survival, we focused on MEX3A because it was recently shown to mark an intestinal stem cell population that is refractory to chemotherapeutic treatments, a typical feature of PDAC. Increased expression of MEX3A was correlated with higher disease stage in PDAC patients and with tumor development in a mouse model of PDAC. Depletion of MEX3A in PDAC cells enhanced sensitivity to chemotherapeutic treatment with gemcitabine, whereas its expression was increased in PDAC cells selected upon chronic exposure to the drug. RNA‐sequencing analyses highlighted hundreds of genes whose expression is sensitive to MEX3A expression, with significant enrichment in cell cycle genes. MEX3A binds to its target mRNAs, like cyclin‐dependent kinase 6 (CDK6), and promotes their stability. Accordingly, knockdown of MEX3A caused a significant reduction in PDAC cell proliferation and in progression to the S phase of the cell cycle. These findings uncover a novel role for MEX3A in the acquisition and maintenance of chemoresistance by PDAC cells, suggesting that it may represent a novel therapeutic target for PDAC.

## Graphical abstract

RNA dysregulation is a hallmark of most human cancers, including PDAC. We identify the RNA‐binding protein MEX3A as prognostic factor in PDAC. MEX3A regulates stability of transcripts for cell cycle proteins, such as CDK6, and promotes cell cycle progression and resistance to chemotherapeutic treatments.

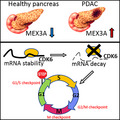

**Abbreviations**
CLIP: UV‐crosslink and RNA immunoprecipitation
DFS: disease‐free survival
DR: drug resistant
EMT: mesenchymal transition
MC: MITO‐Cre
MKC: MITO‐Kras‐Cre
PARG: poly (ADP‐ribose) glycohydrolase
PDAC: pancreatic ductal adenocarcinoma
PI: propidium iodide
RBPs: RNA‐binding proteins
RNA‐seq: RNA sequencing
RNP: ribonucleoprotein
TGCA: The Cancer Genome Atlas

## 1 Introduction

Pancreatic ductal adenocarcinoma (PDAC) is one the most lethal human tumors, with 5‐year survival rate < 9% [1, 2]. Late diagnosis and/or early development of metastases strongly limit therapeutic options for PDAC patients. Despite advances in surgery and chemotherapy, prognosis has only slightly improved over the past decade. Thus, identification of early biomarkers and development of novel therapies represent clinical priorities for this disease [3].

Recent genome‐wide analyses of cancer cell transcriptomes have shown profound dysregulation of gene expression programs, particularly at the level of RNA processing [4, 5]. Cancer cells often express transcript variants that are not present in normal cells, which can generate protein isoforms conferring growth advantage over the host tissue [5]. In some cases, dysregulation of RNA metabolism in cancer cells is associated with somatic mutations in the target genes [6]. However, aberrant expression or activity of regulatory RNA‐binding proteins (RBPs) can also alter RNA metabolism in cancer [7]. Most RBPs recognize specific sequences in the RNA and contribute to regulation of the human transcriptome. They generally act cooperatively by forming dynamic ribonucleoprotein (RNP) complexes, which modulate nuclear RNA processing and export to the cytoplasm, as well as stability, translation, and decay of mature transcripts in the cytoplasm [8, 9]. Thus, changes in the expression or activity of RBPs can directly lead to genome‐wide changes in the cell transcriptome and proteome, which impact on oncogenic features and chemoresistance of cancer cells [10, 11].

Dysregulation of RNA metabolism can be also induced by chemotherapeutic treatments, hence supporting evolution of the tumor toward more resistant phenotypes [12]. In the case of PDAC, it was shown that gemcitabine, one of the first‐line drugs for this cancer, induces the expression of two multifunctional RBPs, SRSF1 and PTBP1, which in turn enhance survival to chemotherapeutic treatments through alternative splicing regulation. Short‐term exposure of PDAC cells to gemcitabine induces the expression of SRSF1 and splicing of the pro‐oncogenic MNK2b variant [13]. Moreover, chronic treatment with this drug caused the selection of clones that overexpressed PTBP1, which promotes aerobic glycolysis through splicing of the PKM2 isoform of pyruvate kinase [14]. Chemotherapy response could also be modulated by alternative polyadenylation of selected transcripts. The transcription factor ZEB1 is a master regulator of epithelial to mesenchymal transition (EMT) that is associated with resistance to chemotherapy and with poor prognosis in PDAC [15]. Treatment of PDAC cells with gemcitabine caused shortening of the 3′ untranslated region by alternative polyadenylation. This regulation abolished ZEB1 translational repression by microRNAs, thus yielding higher protein levels [16]. Notably, enhanced PDAC tumorigenicity by loss of microRNA control through alternative polyadenylation has been recently observed on genome‐wide scale by analysis of a cohort of 148 PDAC patients [17], supporting the pathological relevance of this process in the disease. Lastly, control of mRNA stability by specific RBPs also contributes to PDAC growth and progression. For instance, treatment of PDAC cells with PARP inhibitors (PARPi), which are approved as maintenance treatment of patients with BRCA‐mutated metastatic PDAC after response to platinum‐based chemotherapy [18], was shown to induce the cytoplasmic translocation of HuR. In the cytoplasm, HuR stabilizes the poly (ADP‐ribose) glycohydrolase (PARG) mRNA and enhances DNA repair. On the contrary, silencing of HuR increases the efficacy of PARPi in PDAC cell and mouse models [19]. These observations highlight the relevance of changes in RBP expression for PDAC tumorigenesis. Nevertheless, a comprehensive view of the impact of RBPs on PDAC prognosis is still lacking.

Herein, to evaluate the prognostic value of RBP expression in PDAC, we carried out a bioinformatics screening of The Cancer Genome Atlas (TGCA) database. Out of 12 genes that were associated with disease‐free survival (DFS) in PDAC patients, we focused on MEX3A because its expression was associated with a subpopulation of intestinal stem cells that is refractory to chemotherapeutic treatments [20]. Furthermore, it was recently reported that high expression of MEX3A correlates with advanced PDAC stage and worse prognosis in patients, indicating its oncogenic function in this cancer [21]. Accordingly, we found that MEX3A expression correlates with resistance to gemcitabine in PDAC cells and with pancreatic tumorigenesis in PDAC patients and mice. RNA‐sequencing analysis of MEX3A‐depleted cells identified cell cycle progression as a functional process regulated by this RBP. Our data show that MEX3A promotes the expression of CDK6, a kinase required for the G1‐S transition with strong therapeutic relevance for PDAC [22], while MEX3A depletion significantly impairs S phase entry and cell proliferation. These findings suggest that MEX3A represents a key oncogenic factor and new potential therapeutic target for PDAC treatment.

## 2 Materials and methods

### 2.1 Cell culture, transfections, and treatments

MiaPaCa‐2, ASPC1, C5M2 cells were cultured in DMEM medium (Sigma D5796, Darmstadt, Germany) supplemented with 10% fetal bovine serum (Gibco, Waltham, MA, USA), Non‐Essential Amino Acids 100× (Thermo Fisher 11140035, Waltham, Massachusetts, USA), and gentamicin 200× (Thermo Fisher 15710‐049). HPAF‐II and Pt45P1 were cultured in RPMI 1640 medium (Euroclone ECM2001L, Milan, Italy) supplemented with 10% fetal bovine serum, Non‐Essential Amino Acids 100×, and gentamicin 200×. Cells were grown in a 37 °C humidified atmosphere of 5% CO_2_.

To generate drug‐resistant (DR) clones, we exposed MiaPaCa‐2 cells to treatment with gemcitabine (1 μm) for 7 days. At the end of treatment, surviving clones were amplified and recurrently treated with the drug to maintain the selection. The Pt45P1‐DR clones were generated previously in our laboratory [14].

MiaPaCa‐2 cells were transfected with FLAG or FLAG‐MEX3A plasmids using Lipofectamine 2000 (Invitrogen, Carlsbad, California, USA). For RNAi, cells were transfected with 100 nm siRNAs using Lipofectamine RNAi Max (Invitrogen) and Opti‐MEM medium (Invitrogen). SiRNA for MEX3A (siMEX3A‐1) are sense 5′‐GUGUUUCCCUUCACUCUCUdTdT‐3′ and antisense, 5′‐AGAGAGUGAAGGGAAACACdTdT‐3′ (Sigma), whose sequences are taken from paper of Jiang H. [23] MEX3A On target plus smartpool siRNAs (siMEX3A‐sp) were purchased from Dharmacon (Lafayette, Colorado, USA).

MiaPaCa‐2 cells were synchronized by incubation with 0.5 mm mimosine (Sigma) for 18 h [24]. Cells were then washed three times with PBS and collected at the times indicated in figures. Gemcitabine (Eli Lilly & Company, Indianapolis, IN, USA) was dissolved in water and stored at –20 °C for up to one month.

### 2.2 Mouse strains

All animal studies were approved by the Institutional Animal Care of the Regina Elena National Cancer Institute and by the Government Committee of National Minister of Health and were conducted according to EU Directive 2010/63/EU for animal experiments. LSL‐Kras^G12D/+^ mice and Pdx‐1‐Cre transgenic mice [25, 26] were interbred with FVB MITO‐Luc reporter mice [27] to obtain MITO; LSL‐KrasG12D/+; Pdx‐1‐Cre. The LSL‐KrasG12D/+ lineage was maintained heterozygous state [28].

After genomic DNA extraction of tail biopsies, positive founder animals were identified by PCR using primers specific for the transgenes (Table S1).

### 2.3 Bioluminescence imaging

Mice (24–28 weeks old) were anesthetized, and 75 mg·kg^−1^ of d‐luciferin (Caliper, Perkin Elmer, Waltham, Massachusetts, USA) was injected intraperitoneally. Ten minutes later, quantification of light emission was acquired for 5 min. Signal was detected using the IVIS LuminaII CCD camera system and analyzed with the Living Image 2.20 software package (Caliper Life Sciences, Waltham, Massachusetts, USA). Photon emission was measured in specific regions of interest (ROIs). Data were expressed as photon·second^−1^·cm^−2^·steradiant^−1^ (p·s^−1^·cm^−2^·sr^−1^). After sacrifice, pancreas was resected and analyzed for bioluminescence imaging (BLI) *ex vivo*. The intensity of bioluminescence was color‐coded for imaging purposes; the scale used in the experiment is reported in the figures.

### 2.4 Bioinformatics analysis

Analysis of RBPs associated with PDAC prognosis was performed by screening ‘The Cancer Genome Atlas (TCGA)’ database. Expression of mRNAs detected by the RNA‐sequencing analysis of Pancreatic Adenocarcinoma (TCGA, Firehose Legacy) was downloaded from cBioPortal (https://www.cbioportal.org/study/summary?id=paad_tcga). Disease‐free survival (DFS) was evaluated by using Kaplan–Meier analysis and multivariate Cox proportional hazard regression model. The log‐rank test was used to assess differences between curves. Patients with high signal intensity and low signal intensity were defined by considering positive and negative *z*‐score values. Significance was defined at the *P* < 0.05 level. Analyses were performed with matlab R2019 or cbioportal tool (https://www.cbioportal.org/study/summary?id=paad_tcga). Annotation of high‐purity PDAC samples in basal or classic subtype was retrieved from TCGA Research Network [29]. Analysis for differential expression of RBPs between these subtypes was performed using the visual interface of the psichomics r package [30]. Correlation of MEX3A expression with markers of either basal or classic PDAC subtype was performed using the cBioPortal database. Association of RBP expression with PDAC stage was analyzed with the R2 genomics platform (http://r2.amc.nl – Tumor Pancreatic adenocarcinoma ‐TCGA‐178‐rsem‐tcgars).

### 2.5 RT‐PCR and qPCR analysis

Cellular total RNA was extracted using TRIzol reagent (Invitrogen Thermo Fisher) according to the manufacturer's instructions. After digestion with RNase‐free DNase (Thermo Fisher), RNA was resuspended in RNase‐free water (Sigma‐Aldrich) and retrotranscribed (1 μg) using M‐MLV reverse transcriptase (Promega, Madison, Wisconsin, USA). For extraction of RNA extract from murine pancreas, the tissue was immediately immersed in RNAlater (Thermo Fisher) and frozen. After thawing, tissue was mechanically homogenized by using the dispergierstation T8.10 (IKA WERKE, Staufen im Breisgau, Germany) and RNA was isolated with RNeasy Mini Kit (Qiagen) and treated with RNase‐Free DNase Set (Qiagen, Hilden, Germany). Twenty nanogram of cDNA was used as template for both conventional PCR analysis (GoTaq, Promega) and quantitative real‐time PCR (qPCR) analysis (SYBR Green, Roche, Basel, Switzerland). Primers used for PCR reactions are listed in Table S1.

### 2.6 Protein extraction and western blot

The pellet of cells was resuspended in lysis buffer (150 mm NaCl, 15 mm MgCl_2_, 15 mm EDTA, 50 mm Hepes, 10% Glycerol, 20 mm β‐glicerophosphate, 1% Triton X‐100). Pancreatic tissue was extracted by RIPA buffer (Tris/HCl 50 mm, NP40 1%, NaCl 150 mm, Na‐Deoxycholate 0.5%, EDTA 2 mm, SDS 0.1%). Both buffers were completed with 1 mm dithiothreitol, 2 mm Na‐orthovanadate, and Protease Inhibitor Cocktail (Sigma). After 10 min of incubation on ice, extracts were sonicated at maximum intensity for 5 s and centrifuged for 10 min at 12 000 g at 4 °C. Supernatant fractions were resuspended in SDS/PAGE sample buffer and boiled for 10 min.

For nuclear and cytoplasmic fractionation, cell pellets were resuspended in modified lysis buffer (50 mm Tris/HCl pH 7.5, 0.5% Triton X‐100, 137.5 mm NaCl, 10% Glycerol, 1 mm Na‐orthovanadate, 50 mm Na‐fluoride, 10 mm Na‐pyrophosphate, 5 mm EDTA, and Protease Inhibitor Cocktail). After incubation on ice for 15 min, insoluble nuclei were separated by centrifugation at 13 000 r.p.m. for 15 min at 4 °C and the supernatant (cytoplasmic fraction) collected. After rinsing once with the lysis buffer, the nuclear pellet was resuspended in the lysis buffer containing 0.5% SDS and released genomic DNA sheared by sonication for 5 s at maximum intensity. Then, extracts were centrifuged for 10 min at 12000 g at 4 °C, collect the supernatant (nuclear fraction). Supernatants were resuspended in SDS/PAGE sample buffer and boiled for 10 min.

Western blot analyses were performed as previously described [31] using the following primary antibodies (overnight at 4 °C): rabbit anti‐MEX3A (1 : 1000, HPA062703 Sigma), rabbit anti‐ALDH1A3 (1 : 1000, AP7847A Abgent, San Diego, California, USA), rabbit anti‐CDK6 (1 : 1000, D4S8S Cell Signaling, Carlsbad, California, USA), goat anti‐PTBP1 (1 : 1000, sc‐16547 Santa Cruz Biotechnology), mouse anti‐Actin (1 : 1000, sc‐47778 Santa Cruz Biotechnology, Dallas, Texas, USA), and mouse anti‐GAPDH (1 : 1000, SC‐32233 Santa Cruz Biotechnology). Secondary anti‐mouse or anti‐rabbit IgGs conjugated to horseradish peroxidase (Amersham, Little Chalfont, UK) were incubated for 1 h at RT (1 : 10 000). Immunostained bands were detected by chemiluminescence method (Bio‐Rad, Hercules, California, USA).

### 2.7 Cell viability assays

For MTS assay (Promega), cells were plated at 50% confluence in 96 wells, and after 72 h of treatment, the cell viability was evaluated by assessing the optical density (OD) at 490 nm following the manufacturer's instructions.

For clonogenic assay, single‐cell suspensions were plated in multiwell‐6 (2000 cells/well). The next day, the cells are treated, and after 10 days, cells were fixed in methanol 100% for 10 min at room temperature, stained overnight with crystal violet 0.05% (Sigma‐Aldrich), washed with H_2_O, and dried. Pictures were taken using a digital camera to count and measure the colonies.

### 2.8 Cell cycle analysis

Cell cycle was evaluated by flow cytometry using single staining with propidium iodide (PI) (20 mg·mL^−1^) or double staining with PI and anti‐BrdU antibody (0.125 μg × sample), in the presence of ribonuclease A (1 μg·mL^−1^) as previously described [32]. All cell cycle phases were established using asynchronized control samples. A total of 10 000 events were counted with BD FACSCanto flow cytometer (Becton Dickinson, Franklin Lakes, New Jersey, USA) and analyzed using flowjo v.10 software (Becton Dickinson).

### 2.9 UV‐crosslinked RNA immunoprecipitation (CLIP) assay

CLIP assays were performed as previously described [33] using the anti‐FLAG antibody (3 μg) for immunoprecipitation 1 mg of cell extracts. RNA associated with FLAG‐MEX3A, or with control immunoprecipitation, was represented as percentage of input.

### 2.10 RNA‐sequencing analysis

For RNA‐sequencing (RNA‐seq) analysis, total RNA was extracted using Qiagen RNeasy mini plus Kit from MiaPaCa‐2 cells transfected with Ctrl (*n* = 4), MEX3A SP (*n* = 4) siRNA for 48 h.

The libraries were constructed by QuantSeq 3′ mRNA. Differential gene expression was evaluated after filtering out genes whose expression levels were below 25 RPKM. Analysis for enriched Gene Ontology functional clusters was performed using g:Profiler (https://biit.cs.ut.ee/gprofiler/gost) and Enrichr (https://amp.pharm.mssm.edu/Enrichr/).

## 3 Results

### 3.1 Identification of RNA processing regulators associated with PDAC prognosis

**Fig. 1:**
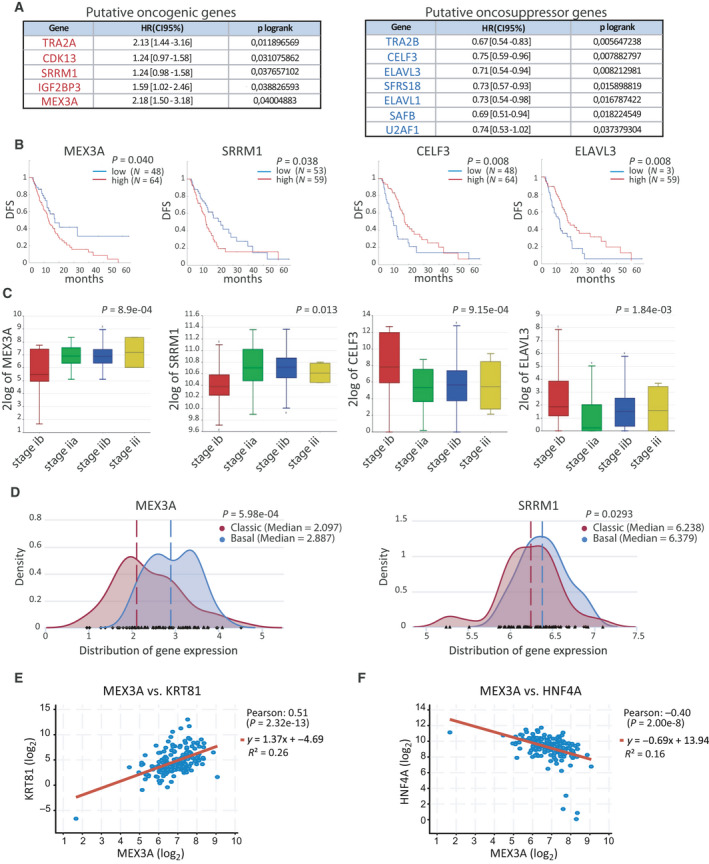
RNA processing regulators associated with PDAC prognosis. (A) RBPs and kinases whose expression is significantly correlated with DFS in PDAC patients annotated in TGCA database. Genes are grouped as potential oncogenes and oncosuppressors on the basis of their association with DFS. HR [CI 95%] indicates the hazard risk of COX regression. (B) Kaplan–Meier curve of DFS for MEX3A and SRRM1 (oncogenic‐like factors) and CELF3 and ELAVL3 (oncosuppressor‐like factors) in PDAC patients. (C) Box‐plot analysis displaying correlation between expression levels and disease stage in PDAC patients for ‘oncogenic‐like’ factors (i.e., MEX3A and SRRM1) and ‘oncosuppressor‐like’ factors (CELF3 and ELAVL3). (D) Density plot of MEX3A and SRRM1 gene expression in PDAC patients from TCGA that were classified as basal‐like or classical subtype according to Moffitt's classification. Dotted lanes indicate the median gene expression value in the two groups of patients. (E, F) Scatter plot of Pearson's correlation analysis of MEX3A and KRT81 (E) or HNF4A (F) performed using the cBioPortal database. Statistical analyses were performed by multivariate Cox proportional hazard regression model (A, B), one‐way ANOVA (C) and Welch two‐sample *t*‐test (D).

To search for RNA processing regulatory factors that may exert an impact on PDAC biology, we performed an unbiased bioinformatic screening by querying The Cancer Genome Atlas (TCGA) database for genes associated with disease outcome in surgically resected PDAC patients. We selected 204 genes encoding RBPs and other proteins (i.e., kinases) that are involved in RNA processing (i.e., splicing and/or cleavage and polyadenylation, translation) (Table S2). Among them, only 12 genes displayed a significant association with DFS of PDAC patients. In some cases, high expression of the RBP associated with worse DFS (i.e., MEX3A and SRRM1), suggesting their potential ‘oncogenic‐like’ function (Fig. 1A,B). By contrast, other RBPs resembled ‘oncosuppressors’, as their high expression levels associated with better prognosis (i.e., CELF3 and ELAVL3), (Fig. 1A,B). Moreover, analysis of another PDAC dataset (http://r2.amc.nl – Tumor Pancreatic adenocarcinoma‐TCGA‐178‐rsem‐tcgars) indicated that ‘oncogenic‐like’ factors (i.e., MEX3A and SRRM1) display positive correlation with higher disease stage, whereas the opposite trend was observed for ‘oncosuppressor‐like’ factors (CELF3 and ELAVL3) (Fig. 1C). These observations suggest that PDAC progression and outcome correlates with, and may be affected by, a selected repertoire of RNA processing factors.

Recent genome‐wide sequencing analyses have indicated the existence of two PDAC subtype, named classical and basal‐like [34]. Basal‐like tumors are characterized by a more aggressive clinical behavior with respect to the classical subtype [28, 29, 31, 34, 35, 36], as also shown by DFS analysis (Fig. S1A). Transcriptomic analysis of PDAC samples that were screened for homogenous cancer cell population [29] using the Psycomics tool [30] indicated that MEX3A, and to a lesser extent SRRM1, were expressed at significantly higher levels in basal‐like tumors (Fig. 1D). Accordingly, MEX3A expression was positively correlated with markers of basal‐like tumors [37], such as KRT14 and KRT81 (Fig. 1E, Fig. S1B), whereas negative (HNF4) or no correlation (GATA6) was found with two markers of classical tumors (Fig. 1F, Fig. S1C). These observations further correlate MEX3A expression with poor prognosis in PDAC.

### 3.2 MEX3A expression is associated with resistance to gemcitabine in PDAC cells

**Fig. 2:**
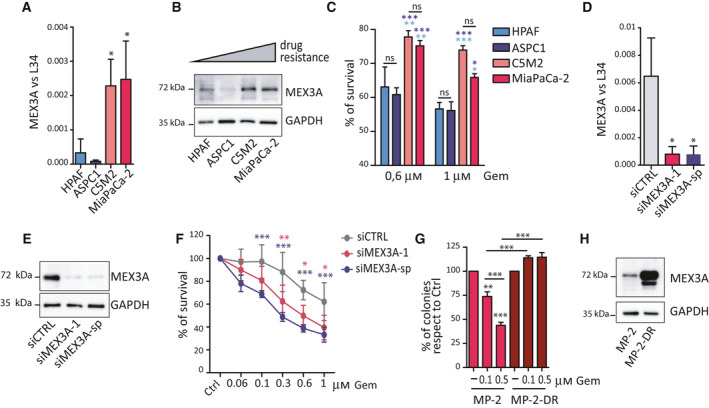
MEX3A promotes resistance to gemcitabine in PDAC cells. (A, B) Analyses of MEX3A expression in PDAC cells by qPCR (A) and western blot (B). Data were normalized for L34 (A) and GAPDH (B) expression. (C) Percentage of survival evaluated by MTS assay after 72 h of treatment with two doses of gemcitabine (0.6 and 1 μm) in PDAC cells. Statistical analyses are reported in light blue when is referred to HPAF cells, while in blue when is referred at ASPC1 cells. (D, E) Analyses of MEX3A expression by qRT‐PCR (D) and western blot (E) to evaluate depletion of the transcript and protein after silencing with siMEX3A‐1 or smartpool (sp) siRNAs. (F) Percentage of survival evaluated by MTS assay after 72 h of treatment with the indicated doses of gemcitabine in MiaPaCa‐2 cells transfected with CTRL, MEX3A‐1 or MEX3A‐sp siRNAs. (G) Clonogenic assay to evaluate survival of the parental and drug‐resistant (MP‐DR) MiaPaCa‐2 cells in the presence of the indicated doses of gemcitabine. (H) Western blot of MEX3A expression in MP‐2‐DR and parental MiaPaCa‐2 cells. Statistical analyses were performed by one‐way ANOVA (A, D, G) and two‐way ANOVA (C, F). *P ≤ 0.05, **P ≤ 0.01, ***P ≤ 0.001.

Poor prognosis in PDAC relies on rapid acquisition of chemoresistance [3]. MEX3A was reported to mark an intestinal stem cell population that is particularly refractory to chemotherapeutic treatments [20]. Moreover, MEX3A protein expression was recently associated with poor prognosis in a cohort of PDAC patients [21]. Thus, we focused our study on MEX3A. To test the predictive value of our bioinformatic analysis, we evaluated whether expression of MEX3A correlates with drug resistance in PDAC cell lines. MEX3A was expressed at higher transcript and protein levels in the more malignant C5M2 and MiaPaCa‐2 cells with respect to less aggressive HPAF‐II and ASPC1 cells (Fig. 2A,B). Notably, HPAF‐II cells were described as classical PDAC subtype, whereas MiaPaCa‐2 cells clustered with basal‐like PDAC [37]. We observed that MEX3A was also expressed at much higher level in an another basal‐like cell line (PANC‐1) than in the classical‐like cell line Capan‐1 (Fig. S2A) and that its expression was inversely correlated with that of GATA6 (Fig. S2B). These results further suggest that MEX3A expression is associated with the basal‐like PDAC subtype.

Viability assays using two doses of gemcitabine, a drug frequently included in first‐line chemotherapeutic treatments for PDAC [3], confirmed that cells expressing higher levels of MEX3A are more resistant to treatments than low‐expressing cells (Fig. 2C). To investigate whether MEX3A contributed to drug resistance in PDAC cells, we silenced its expression by RNA interference (RNAi). MEX3A depletion using two different siRNAs significantly enhanced sensitivity to gemcitabine over a range of drug concentrations in both MiaPaCa‐2 (Fig. 2D–F) and C5M2 cells (Fig. S2C,D).

Next, we generated drug‐resistant clones (MiaPaCa‐2‐DR) by exposure of MiaPaCa‐2 cells to treatment with 1 μm gemcitabine for 7 days. At the end of the selection, surviving clones were isolated and amplified under recurring monthly treatments with the drug to maintain the selection. To confirm the phenotype of MiaPaCa‐2‐DR cells, we analyzed cell survival by clonogenic assays. While exposure to gemcitabine reduced the number of colonies in a dose‐dependent manner in parental MiaPaCa‐2 cells, DR cells were insensitive to treatments (Fig. 2G). Importantly, MEX3A expression was further increased in MiaPaCa‐2‐DR cells with respect to parental cells (Fig. 2H). Furthermore, a similar up‐regulation of MEX3A was also observed in another clone of drug‐resistant PDAC cells that we previously established (Pt45P1‐DR; Fig. S2E) [14]. These results support a functional role for MEX3A in the acquisition and maintenance of drug resistance in PDAC cells.

### 3.3 MEX3A expression is increased in a mouse model of PDAC

**Fig. 3:**
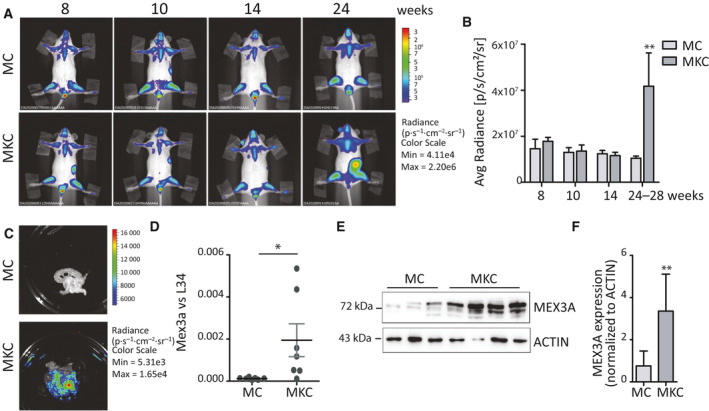
MEX3A expression is increased in a mouse model of PDAC. (A) Longitudinal monitoring of PDAC‐associated proliferation by *in vivo* BLI. Representative images of representative MKC and control MC mice were taken at the indicated weeks. (B) Bar graph showing the mean of the abdominal bioluminescence of MKC (*n* = 3) and MC (*n* = 3) mice at the indicated experimental time points. (C) Representative *ex vivo* BLI image of the isolated pancreas from MC and MKC mice shown in panel B. (D, E) Analysis by qPCR (D) and western blot (E) to evaluate MEX3A expression level in MKC and MC mice. (F) Densitometric analysis of MEX3A protein expression levels (*n* = 5 for MC mice and *n* = 9 for MKC mice). Data were normalized for Actin intensity. Statistical analyses were performed by Student's *t*‐test (C–F). *P ≤ 0.05.

Activating mutations in *KRAS* represents the most common genetic driver of PDAC, which is found in up to 90% of patients [38]. Moreover, introduction of mutations in *Kras* is sufficient to cause tumorigenesis in mice [25]. To test whether MEX3A expression was modulated during neoplastic transformation of pancreatic cells, we employed the LSL‐Kras^G12D/+^ Pdx‐1‐Cre PDAC mouse model, which is characterized by conditional expression of an oncogenic *Kras* mutant allele in the pancreas [26]. In particular, we used a mouse model crossed with the MITO‐Luc mouse, which expresses the luciferase reporter gene under the control of a mitotic promoter. In the resulting MITO‐Kras‐Cre (MKC) mice, physiological and aberrant cell proliferation in the organ can be measured by noninvasive bioluminescence imaging (BLI) (Fig. 3A) [28]. Pancreatic tissue was collected from MKC or control mice (MC) at 19–28 weeks of life when abdominal BLI was significantly increased (Fig. 3B), and strong luminescence could be readily detected in the pancreas *ex vivo* (Fig. 3C; Fig. S3A). Quantitative real‐time PCR (qPCR) analysis revealed that MEX3A was barely detectable in normal tissue, whereas its expression was significantly increased in the pancreas isolated from MKC mice (Fig. 3D). Moreover, western blot analyses of pancreatic tissue from MKC mice indicated that MEX3A protein is up‐regulated in the pancreas that has developed a tumor with respect to control mice (Fig. 3E‐F; Fig. S3B). These observations suggest that MEX3A may contribute to PDAC tumorigenesis.

### 3.4 MEX3A regulates genes involved in cell cycle progression

MEX3A has been previously involved in the stability of specific transcripts [39]. However, no information is currently available on the genome‐wide impact of MEX3A on the cell transcriptome. To investigate its functional role in PDAC cells, we performed RNA‐sequencing (RNA‐seq) analysis of MiaPaCa‐2 cells depleted of MEX3A. RNA was extracted after 48 h, when MEX3A was efficiently down‐regulated at transcript level (Fig. 4A) and used for the RNA‐seq analysis. By screening for transcripts displaying ≥ 25 RPKM and fold change ≥ 2, we identified 726 genes that were significantly regulated (*P* < 0.05) by depletion of MEX3A in MiaPaCa‐2 cells. The majority of the MEX3A‐sensitive genes (484, 65%) were down‐regulated (Fig 4B, Table S3). Functional clustering analysis identified ‘cell cycle’, ‘Hippo signalling’, and ‘TGF‐beta signaling’ as pathways enriched among the down‐regulated genes, whereas up‐regulated genes were more heterogeneous, with only three functional categories related to ‘proteasome’ and ‘pathogenic infections’ being enriched (Fig. 4C). Direct qPCR analysis of the expression of MEX3A‐regulated genes (*TCF7, CDK6, TEAD2, ALDH1A3, PTBP1,* and *SF3A1*) confirmed the results of the RNA‐seq (Fig. 4D), thus validating the bioinformatic analysis. Moreover, western blot analysis of PTBP1, ALDH1A3 and CDK6 showed concomitant reduction at the protein level (Fig. 4E), whereas all these targets were expressed at higher level in DR‐MiaPaCa‐2 cells (Fig. S4). Importantly, analysis of pancreas from MKC mice documented increased expression of two of these MEX3A target genes (i.e., *Ptbp1* and *Aldh1a3*) at both mRNA and protein levels with respect to tissues from control mice (Fig. 4F,G).

**Fig. 4(Panel B of this figure has been altered: the numbers in the pie sector should be centered; moreover, the legend is partially hidden by the pie. Please see the original Figure 4 that we have attached here for reference):**
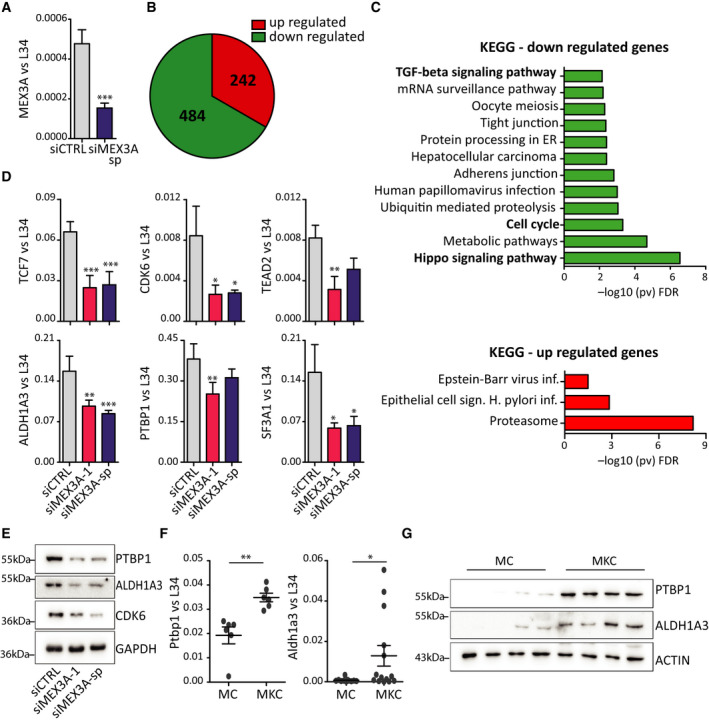
MEX3A regulates genes involved in cell cycle progression in PDAC cells. (A) Analysis by qRT‐PCR of MEX3A expression in MiaPaCa‐2 cells transfected with MEX3A‐sp siRNAs or control siRNAs and used for the RNA‐seq experiment (*n* = 4). (B) Pie chart of genes that are up‐ and down‐regulated in MiaPaCa‐2 cells depleted of MEX3A identified by the RNA‐seq analysis. (C) Gene ontology (KEGG) of up‐ and down‐regulated genes in MiaPaCa‐2 cells depleted of MEX3A performed by using the g‐profiler web server. (D) Validation by qRT‐PCR of the effect of MEX3A depletion on selected target genes. MEX3A depletion was performed by using two separate siRNA sets (siMEX3A‐1 and siMEX3A‐sp). (E) Western blot analysis of the expression of MEX3A targets (PTBP1, ALDH1A3 and CDK6) in MiaPaCa‐2 cells. (F, G) Analysis by qPCR (F) and western blot (G) of the expression of MEX3A target genes (*Ptbp1* and *Aldh1a3*) in MKC and MC mice. Statistical analyses were performed by Student's *t*‐test (A–F) one‐way ANOVA (D). *P ≤ 0.05, **P ≤ 0.01, ***P ≤ 0.001.

### 3.5 MEX3A binds its target transcripts and regulates their stability

**Fig. 5:**
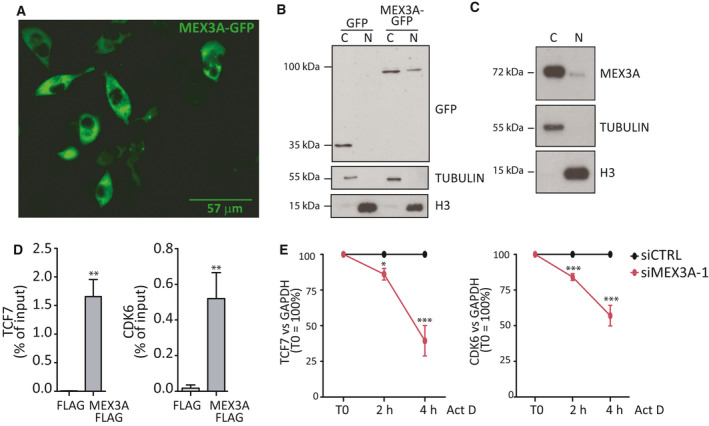
MEX3A binds its target transcripts and regulates their stability. (A) Immunofluorescence analysis of the MEX3A‐GFP recombinant protein; scale bar = 57 μm. (B, C) Subcellular fractionation analyses of recombinant MEX3A‐GFP protein (B) and endogenous MEX3A (C). (D) Bar graphs showing the results CLIP assays performed by anti‐FLAG immunoprecipitates of extracts from MiaPaCa‐2 cells transfected with either empty FLAG plasmid or FLG‐MEX3A plasmid. Data represent qPCR analyses of TCF7 and CDK6 mRNAs co‐precipitated in CLIP experiments and are normalized as percentage of input. (E) TCF7 and CDK6 transcript stability after depletion of MEX3A and treatment with Actinomycin D for the indicated time. The qPCR analyses were normalized to GAPDH mRNA levels and by setting the T0 value as 100%. Statistical analyses were performed by Student's *t*‐test (D) and two‐way ANOVA (E). *P ≤ 0.05, **P ≤ 0.01, ***P ≤ 0.001.

Dysregulation of the Hippo pathway plays a key role in PDAC tumorigenesis [40], whereas inhibition of the cell cycle kinases CDK4 and CDK6 represents a promising therapeutic approach for PDAC [22]. Thus, genes that require MEX3A for their expression play oncogenic functions in PDAC. To further investigate how MEX3A regulates its target transcripts, we first evaluated its subcellular distribution in MiaPaCa‐2 cells. MEX3A was reported to shuttle between nucleus and cytoplasm in other cell types [41]. Transfection of the recombinant protein MEX3A‐GFP indicated that MEX3A is predominantly cytoplasmic in MiaPaCa‐2 cells (Fig. 5A). Subcellular fractionation analyses confirmed this result and indicated that both the recombinant MEX3A‐GFP protein and the endogenous MEX3A were prevalently localized in the cytoplasm of MiaPaCa‐2 cells (Fig. 5B,C). Thus, we tested whether MEX3A regulates expression of its target transcripts at the post‐transcriptional level.

First, we asked if MEX3A directly interacts with its target mRNAs. To this end, we performed UV‐crosslink and RNA immunoprecipitation (CLIP) assay in MiaPaCa‐2 cells over‐expressing FLAG‐MEX3A or transfected with empty vector. A strong enrichment of CLIP signals for TCF7, CDK6, and other MEX3A‐regulated transcripts was detected in the MEX3A immunoprecipitates with respect to control samples (Fig. 5D; Fig. S5A).

To test whether MEX3A modulates the half‐lives of its target transcripts, we performed stability assays in live cells. MiaPaCa‐2 cells were treated with Actinomycin D to block transcription, and decay of transcripts was measured by qPCR at different time points. Importantly, depletion of MEX3A significantly accelerated the decay of CDK6 and TCF7 mRNAs in MiaPaCa‐2 cells (Fig. 5E), whereas it did not affect a control, non‐regulated gene (Fig. S5B). These results suggest that MEX3A regulates gene expression by directly binding to target mRNAs.

### 3.6 MEX3A affects cell cycle progression in PDAC cells

**Fig. 6:**
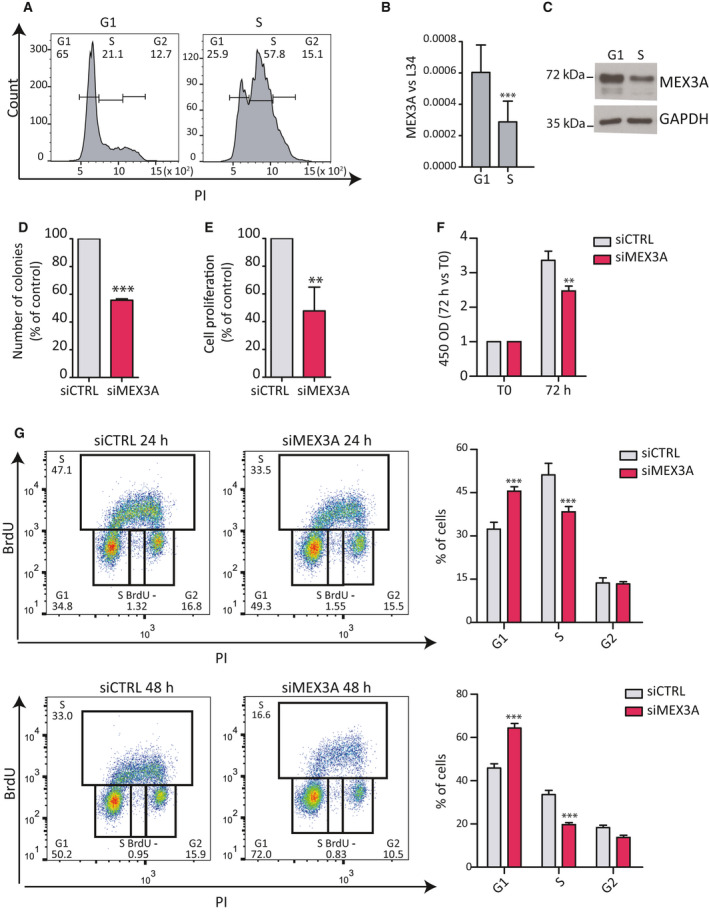
MEX3A regulates cell cycle progression in PDAC cells. (A) FACS analysis showing DNA content (PI) of MiaPaCa‐2 cells after release from mimosine‐induced synchronization. Times were chosen for enrichment of cells in the G1 and S phases of the cycle. (B, C) Analyses by qPCR (B) and western blot (C) of MEX3A expression in G1 and S phase cells. (D) Clonogenic assay performed in MiaPaCa‐2 cells transfected with CTRL or MEX3A siRNAs. Colonies were counted after 10 days from seeding. (E) Analysis of cell proliferation of MiaPaCa‐2 cells transfected with CTRL or MEX3A siRNAs. Data are represented as percentage of growth of control cells after 72 h. (F) MTS assay after of MiaPaCa‐2 cells transfected with CTRL or MEX3A siRNAs. Data are represented as fold increase at 72 h with respect to time of seeding (T0). (G) FACS analysis showing DNA content (propidium iodide, PI) and bromodeoxyuridine (BrdU) incorporation of MiaPaCa‐2 cells transfected with CTRL or MEX3A targeting siRNAs at 24 or 48 h. The percentage of cells in G1, S, and G2 phase are indicated. Statistical analyses were performed by the Student's *t*‐test (B, D, E) or two‐way ANOVA (F, G). *P ≤ 0.05, **P ≤ 0.01, ***P ≤ 0.001.

Regulation of the cell cycle emerged as one of the main pathways regulated by MEX3A in PDAC cells. Thus, we investigated the impact of MEX3A expression on cell cycle progression. Synchronization of MiaPaCa‐2 cells by mimosine‐induced block [24] indicated that MEX3A expression is regulated during the cell cycle, being higher in G1 and decreasing in the subsequent S phase (Fig. 6A–C). Moreover, depletion of MEX3A in MiaPaCa‐2 cells caused a significant reduction in proliferation, as measured by colony formation assay, cell counting and proliferation assay (Fig. 6D–F). Notably, FACS analysis showed that MEX3A depletion causes a significant enrichment in G1 and reduction of S phase already after 24 h, a phenotype that was more dramatic after 48 h from silencing (Fig. 6G).

**Fig. 7:**
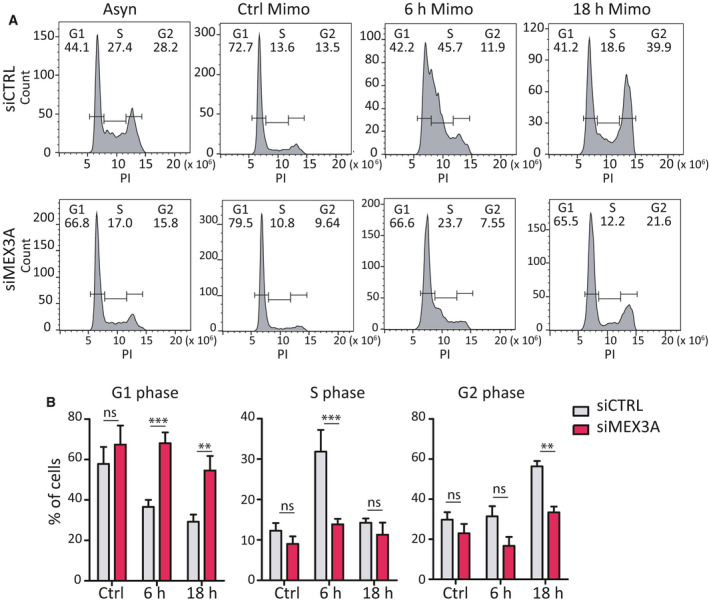
MEX3A acts on transition G1/S phase of cell. (A) FACS analysis showing DNA content (PI) of MiaPaCa‐2 cells after release from mimosine synchronization in cells transfected with CTRL or MEX3A siRNAs. (B) Bar graphs show the percentage of cells in G1, S, and G2 phase of cell cycle by FACS analysis of panels shown in (A). Statistical analyses were performed by two‐way ANOVA (B). **P ≤ 0.01, ***P ≤ 0.001.

The Cyclin D1/CDK6 complex promotes the G1/S transition of the cell cycle [42]. Since CDK6 is reduced in MEX3A‐depleted cells, we tested whether MEX3A was required for S phase entry. Following synchronization in G1 by mimosine treatment, cells were released and cell cycle progression was monitored by FACS analysis. Strikingly, MEX3A‐depleted MiaPaCa‐2 cells remained stalled in the G1 phase after release from the drug, whereas most control cells progressed to the S phase by 6 h and entered in the G2 phase of the cycle by 18 h (Fig. 7A,B). These findings suggest that MEX3A expression is required for the completion of the G1 phase and the transition into the S phase of the mitotic cycle of PDAC cells.

## 4 Discussion

Pancreatic ductal adenocarcinoma has emerged as a clinical priority in oncology in the last decade [1], due to its extremely poor prognosis and refractoriness to therapies [3]. Indeed, PDAC is predicted to become the second cause of death for cancer by 2030 [43]. As previously experienced for other cancers [44, 45], it is likely that a better understanding of PDAC biology will be instrumental for the identification of new therapeutic targets and for the development of innovative therapies that counteract acquisition of chemoresistance. In this study, we show that MEX3A is up‐regulated in PDAC patients and associates with poor prognosis. MEX3A binds to and modulates the expression of hundreds of mRNAs in PDAC cells, particularly those encoded by cell cycle genes. Moreover, MEX3A promotes resistance to gemcitabine, which is frequently used either alone or in combination with nab‐paclitaxel as first‐line chemotherapeutic treatment for PDAC, suggesting a direct role for this RBP in the acquisition of a drug‐resistant phenotype.

Aberrant expression of RBPs is a common phenomenon during development and progression of human cancers. Dysregulated RBPs often promote oncogenic isoforms that affect cell cycle regulation, cell proliferation, and invasion [46]. Our bioinformatic screen aimed at identifying RBPs associated with disease outcome in PDAC yielded only 12 out of 204 genes with significant correlation, of which only five were associated with poor prognosis. This relatively small number of genes may be due to the small cohort of PDAC patients (*n* = 112) for which full data are available in the TCGA database. Nevertheless, to validate the reliability of our predictive analysis we focused on MEX3A, because this RBP was recently shown to mark intestinal stem cells that are refractory to chemotherapeutic treatments [20]. This observation attracted our attention because subpopulation of cancer cells that are particularly resistant to chemotherapeutic treatments are thought to guide relapse of disease in many cancers, including PDAC [47]. Moreover, a recent screening of 1542 human RBPs dysregulated across 15 cancer types indicated that MEX3A is up‐regulated in multiple epithelial cancers [46]. Nevertheless, relatively little is known on the oncogenic functions of MEX3A and even less on the molecular mechanisms modulated by this RBP in cancer cells. Up‐regulation of MEX3A in gastric tumor tissues was previously shown to promote cell proliferation and migration, but the mechanism involved was not investigated [23]. Furthermore, while this work was in progress, another study reported the up‐regulation of MEX3A protein in PDAC patients correlated with poor prognosis, whereas depletion of MEX3A in PDAC cells inhibited proliferation and migration *in vitro* and tumor development in mouse xenograft model [21]. However, no insight into the mechanism through which MEX3A elicited these effects was provided. Herein, by using two different PDAC cell lines, we confirmed the effect of MEX3A expression on proliferation and colony formation. We observed that MEX3A transcript and protein levels are increased in PDAC cells displaying a basal‐like phenotype, which has been associated with worse prognosis in PDAC patients [33, 34, 35]. Moreover, MEX3A was up‐regulated *in vivo* in a mouse model of pancreatic tumorigenesis. Our transcriptomic analysis has also identified hundreds of genes whose expression is susceptible to MEX3A levels in PDAC cells. Notably, we found a significant enrichment in cell cycle and Hippo pathway genes, two functional categories of relevance for PDAC biology [20, 29]. Interestingly, MEX3A promoted the expression of genes involved in these pathways also in stem cells of the intestinal crypts [48], corroborating our results. It is also worth mentioning that MEX3A appears essential for the maintenance of intestinal stem cells *in vivo* [39]. This observation, together with the similarity in genes regulated by this RBP in intestinal stem cells and PDAC cells, suggests that high expression of MEX3A may confer stem‐like features to PDAC cells, which are known to be associated with refractoriness to chemotherapeutic treatments. Our data are in line with this hypothesis, as depletion of MEX3A increases susceptibility to gemcitabine, whereas its expression is up‐regulated upon chronic exposure to the drug. These results suggest that targeting MEX3A may represent a valuable approach to improve the efficacy of the current chemotherapeutic treatments for PDAC.

We found that MEX3A is prevalently localized in the cytoplasm of PDAC cells, binds directly to its target transcripts, and promotes their stability. This action on the stability of transcripts has also been found for MEX3B, a member of the MEX3 family with high structural homology to MEX3A. MEX3B was shown to bind the 3′ untranslated region of the BIM mRNA, thus competing with access to miRNAs and leading to up‐regulation of BIM expression and induction of cell death [49]. Binding of MEX3A to its target transcript may also underlie its effect of the phenotype of PDAC cells. For instance, we observed that MEX3A binds to and stabilizes CDK6 mRNA levels. CDK6 is a cyclin D‐associated kinase that promotes the transition between G1 and S phase of the cell cycle. CDK6 expression levels are significantly reduced in MEX3A‐depleted PDAC cells, and this effect correlates with a specific defect of MEX3A‐depleted cells to exit from the G1 phase and enter into the S phase. Thus, the RNA‐binding activity of MEX3A may exert a physiological impact on PDAC cell biology, similarly to what was reported for its homolog MEX3B. Importantly, CDK6 is a suitable target for PDAC, as mutations in its physiological inhibitor p16INK4A, encoded by the *CDKN2A* tumor suppressor gene, are the second most frequent genomic aberration in found PDAC patients [3]. Moreover, inhibitors of CDK4 and CDK6 activity, the two kinases that associate with cyclin Ds to drive entry into S phase, have been recently shown to exert a strong effect in PDAC when combined with conventional chemotherapeutic treatments [20]. We now report that MEX3A also promotes the expression of PTBP1, an RBP that was previously shown to contribute to acquisition of a drug‐resistant phenotype in PDAC [14]. These observations suggest that MEX3A may also affect therapeutic treatments of PDAC through the regulation of target genes involved in processes and pathways highly relevant for this disease, such as cell cycle regulation and Hippo pathway.

## 5 Conclusions

In conclusion, our study identifies MEX3A as an RBP involved in PDAC, whose high expression correlates with poor prognosis. Furthermore, our study also indicates that MEX3A is a novel biomarker associated with the basal‐like PDAC subtype. These findings suggest that MEX3A represents a key oncogenic factor and, possibly, a new therapeutic target for PDAC treatment.

## Conflict of interest

The authors declare no conflict of interest.

## Author contributions

VP and CS conceived and designed the project, analyzed and interpreted the data, and wrote the paper; IM acquired the data and interpreted the data; VP, AC, LL, and AM acquired the data; CN and AS bioinformatic analysis; SDA, EP, GP, and GC interpreted the data.

## Supporting information


**Fig. S1.** A) Kaplan‐Meier curve of DFS for basal and classic subtype of pancreatic cancer in PDAC patients. Statistical analyses were performed by log rank test. B‐C) Scatter plot of Pearson's correlation analysis of MEX3A and KRT14 (B) or GATA6 (C) performed using the cBioPortal database.
**Fig. S2.** A) Analysis by qPCR of *MEX3A* (A) and *GATA6* (B) expression levels in PDAC cell lines. C) Analysis by qPCR of the MEX3A expression levels in C5M2 cells transfected with the indicated siRNAs. D) Percentage of survival evaluated by MTS assay after 72h of treatment with different doses of gemcitabine in C5M2 cells transfected with CTRL, MEX3A or MEX3A SP siRNAs. E) Western blot analysis of MEX3A expression in PT45P1‐DR cells. Statistical analyses were performed by one way Anova (A‐B‐C) and two way Anova (D).
**Fig. S3.** A) Ex‐vivo images of the isolated pancreas of MC and MKC mice performed by BLI. B) Western blot analysis of MEX3A protein expression in MKC and MC mice.
**Fig. S4.** Western blot analysis of the expression of MEX3A targets (PTBP1, ALDH1A3 and CDK6) in MP‐2‐DR and parental MiaPaCa‐2 cells.
**Fig. S5.** A) Bar graphs shows the results of qPCR analyses of TEAD2, ALDH1A3, PTBP1 and SF3A1 mRNAs co‐precipitated by FLAG antibody in CLIP experiments. The samples are normalized with respect to input. B) L34 transcript stability after depletion of MEX3A and treatment with Actinomycin D for the indicated time.
**Table S1.** List of PCR primers.
**Table S2.** List of RNA processing genes analyzed.
**Table S3.** List of genes regulated in MEX3A‐depleted cells.
