# Supplementary material for: The RNA‐binding protein MEX3A is a prognostic factor and regulator of resistance to gemcitabine in pancreatic ductal adenocarcinoma
Source: Mol Oncol. 2020 Nov 24;15(2):579–95. doi: 10.1002/1878-0261.12847 (PMC7858117; doi:10.1002/1878-0261.12847)
Supplement: Supplementary file 1 — Fig. S1. A) Kaplan‐Meier curve of DFS for basal and classic subtype of pancreatic cancer in PDAC patients. Statistical analyses were performed by log rank test. B‐C) Scatter plot of Pearson's correlation analysis of MEX3A and KRT14 (B) or GATA6 (C) performed using the cBioPortal database. Fig. S2. A) Analysis by qPCR of MEX3A (A) and GATA6 (B) expression levels in PDAC cell lines. C) Analysis by qPCR of the MEX3A expression levels in C5M2 cells transfected with the indicated siRNAs. D) Percentage of survival evaluated by MTS assay after 72h of treatment with different doses of gemcitabine in C5M2 cells transfected with CTRL, MEX3A or MEX3A SP siRNAs. E) Western blot analysis of MEX3A expression in PT45P1‐DR cells. Statistical analyses were performed by one way Anova (A‐B‐C) and two way Anova (D). Fig. S3. A) Ex‐vivo images of the isolated pancreas of MC and MKC mice performed by BLI. B) Western blot analysis of MEX3A protein expression in MKC and MC mice. Fig. S4. Western blot analysis of the expression of MEX3A targets (PTBP1, ALDH1A3 and CDK6) in MP‐2‐DR and parental MiaPaCa‐2 cells. Fig. S5. A) Bar graphs shows the results of qPCR analyses of TEAD2, ALDH1A3, PTBP1 and SF3A1 mRNAs co‐precipitated by FLAG antibody in CLIP experiments. The samples are normalized with respect to input. B) L34 transcript stability after depletion of MEX3A and treatment with Actinomycin D for the indicated time. Table S1. List of PCR primers. Table S2. List of RNA processing genes analyzed. Table S3. List of genes regulated in MEX3A‐depleted cells. [file MOL2-15-579-s001.pdf]

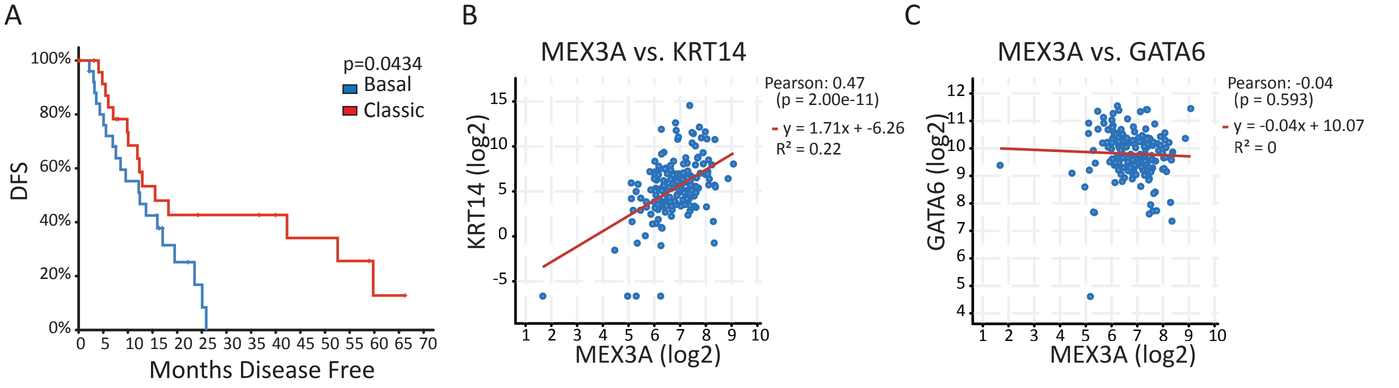

Supplementary Figure 1. A) Kaplan-Meier curve of DFS for basal and classic subtype of pancreatic cancer in PDAC patients. Statistical analyses were performed by log rank test. **B-C)** Scatter plot of Pearson's correlation analysis of MEX3A and KRT14 **(B)** or GATA6 **(C)** performed using the cBioPortal database.

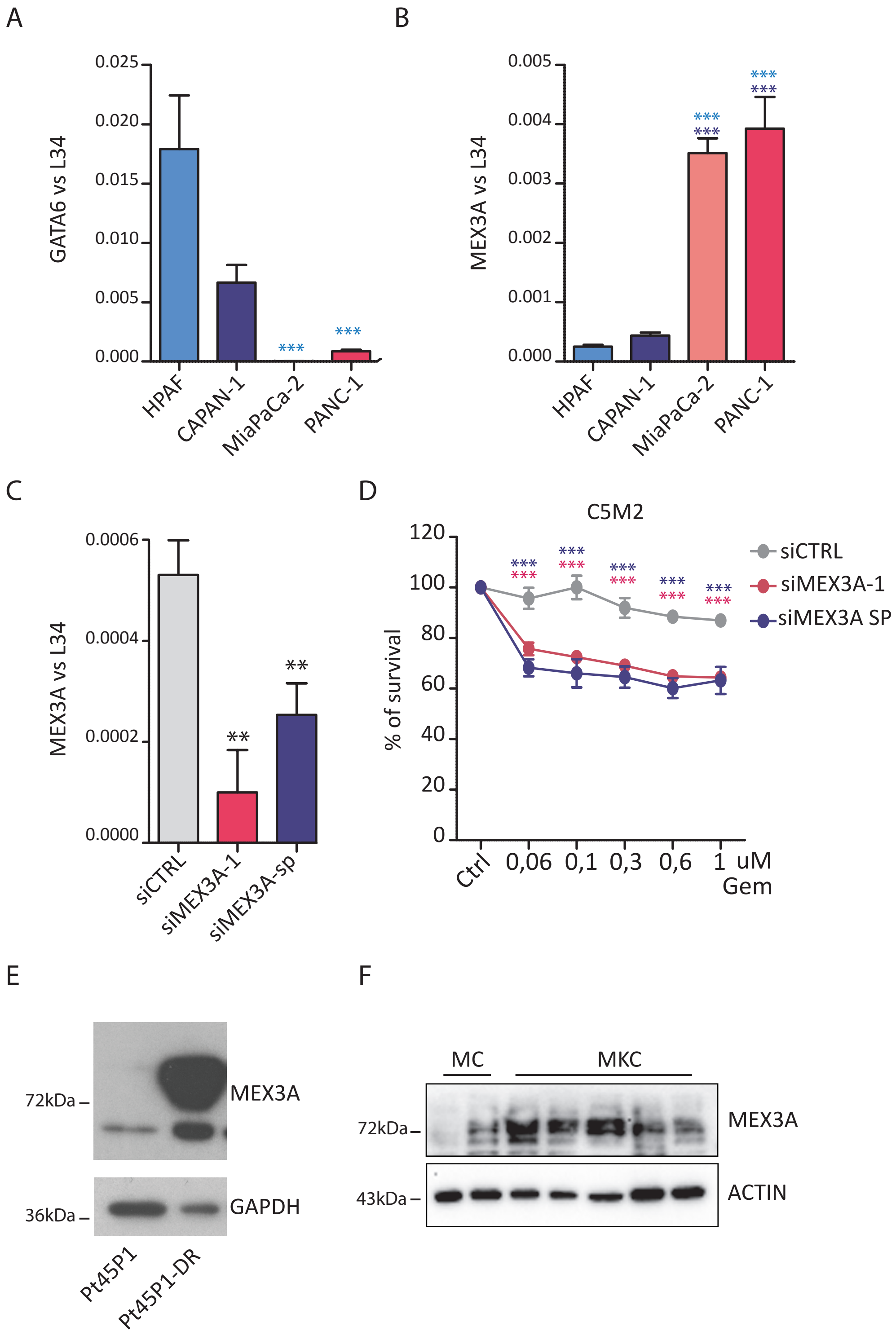

Supplementary Figure 2. A) Analysis by qPCR of *MEX3A* **(A)** and *GATA6* **(B)** expression levels in PDAC cell lines. **C)** Analysis by qPCR of the MEX3A expression levels in C5M2 cells transfected with the indicated siRNAs. **D)** Percentage of survival evaluated by MTS assay after 72h of treatment with different doses of gemcitabine in C5M2 cells transfected with CTRL, MEX3A or MEX3A SP siRNAs**. E)** Western blot analysis of MEX3A expression in PT45P1-DR cells. Statistical analyses were performed by one way Anova **(A-B-C)** and two way Anova **(D)**. ** P≤0.01, ***P ≤ 0.001.

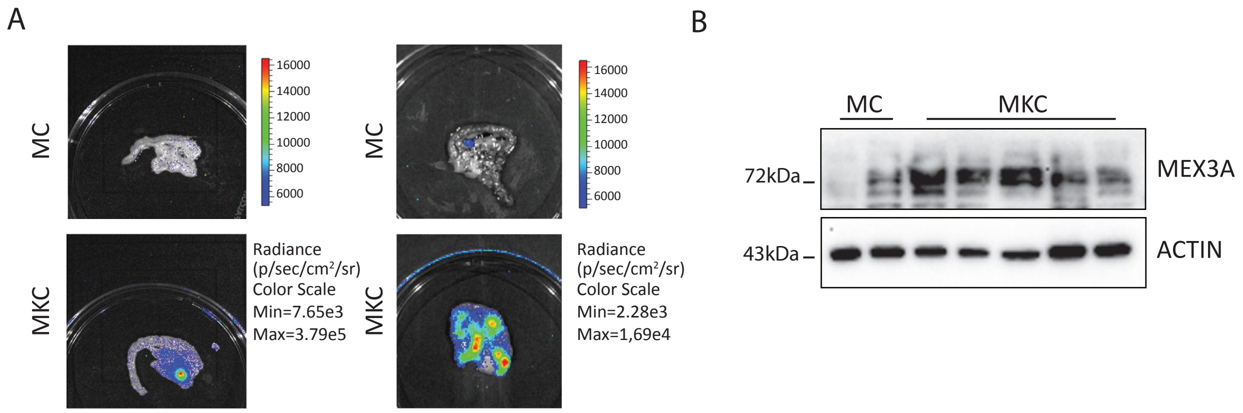

Supplementary Figure 3. A) Ex-vivo images of the isolated pancreas of MC and MKC mice performed by BLI.
B) Western blot analysis of MEX3A protein expression in MKC and MC mice.

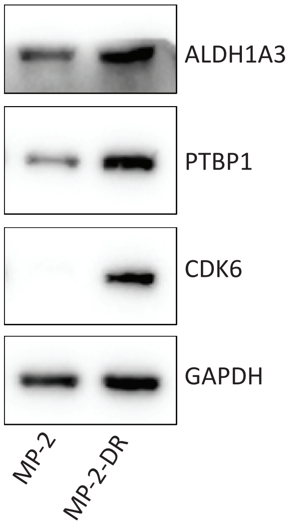

Supplementary Figure 4. Western blot analysis of the expression of MEX3A targets (PTBP1, ALDH1A3 and CDK6) in MP-2-DR and parental MiaPaCa-2 cells.

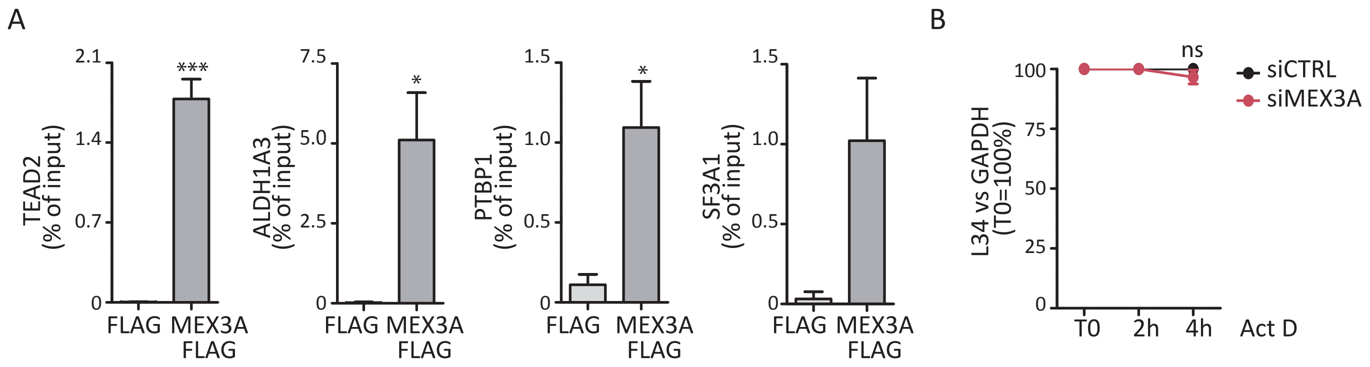

Supplementary Figure 5. A) Bar graphs shows the results of qPCR analyses of TEAD2, ALDH1A3, PTBP1 and SF3A1 mRNAs co-precipitated by FLAG antibody in CLIP experiments. The samples are normalized with respect to input. **B)** L34 transcript stability after depletion of MEX3A and treatment with Actinomycin D for the indicated time. The qPCR data were normalized to GAPDH mRNA levels and the T0 value was set to 100%. Statistical analyses were performed by Student's t-test **(A)** and two way Anova **(B)**. *P ≤ 0.05,***P ≤ 0.001.

Supplementary table 1

List of oligonucleotides used in this study:

hALDH1A fw	GGGCCAGTGCAACCAATACT
hALDH1A rv	TGTGCATAGAGGGCGTTGTAG
mALDH1A fw	TCAACAACGACTGGCACGAA
mALDH1A rv	CCTTGTCCACATCGGGCTTAT
hCDK6 fw	TGACCAGCAGCGGACAAATA
hCDK6 rv	CAAGACTTCGGGTGCTCTGT
hGAPDH fw	CCCTTCATTGACCTCAACTACATG
hGAPDH rv	TGGGATTTCCATTGATGACAAGC
hGATA6 fw	GCCAACTGTCACACCACAAC
hGATA6 rv	CATAGCAAGTGGTCTGGGC
hL34 fw	GTCCCGAACCCCTGGTAATAGA
hL34 rv	GGCCCTGCTGACATGTTTCTT
mL34 fw	GGTGCTCAGAGGCACTCAGGATG
mL34 rv	GTGCTTTCCCAACCTTCTTGGTGT
hMEX3A fw	TCTACAAAGAGGCCGAGCTG
hMEX3A rv	CCCTCACCGGTGTCTTGATG
mMEX3A rv	CCCTCAGAGCCTTAATCTTGC
hPTBP1 fw	GGAAGGTCACCAACCTCCTG
hPTBP1 rv	GGAGAGCTGTCGGTCTTCAG
mPTBP1 fw	GAGGAGGCTGCCAACACTATG
mPTBP1 rv	CGGTCTTGAGCTCTTTGTGGT
hSF3A1 fw	CAGCGAGTTCAAGGAAGGGA
hSF3A1 rv	TGGTCTCTTGGATTACTTGGGC
HTCF7 fw	CCCCCAACTCTCTCTCTACGA
hTCF7 rv	AGGTCAGGGAGTAGAAGCCA
hTEAD2 fw	ACCGCCAGATGCAGTTGATT
hTEAD2 rv	GTCGTAGATCTGCCGGACG
MITO fw	TGTAGACAAGGAAACAACAAAGCCTGGTGGCC
MITO rv	GGCGTCTTCCATTTTACCAACAGTACCGG
Kras fw	GTCTTTCCCCAGCACAGTGC
Kras rv	CTCTTGCCTACGCCACCAGCTC
Kras rv	AGCTAGCCACCATGGCTTGAGTAAGTCTGCA
Pdx-1-Cre fw	ATGCTTCTGTCCGTTTGCCG
Pdx-1-Cre rv	TGAGTGAACGAACCTGGTCG

Supplementary table 2
List of the RNA processing genes analyzed in this study

ACIN1	IGF2BP2	RBM23	SFRS18
AKAP17A	IGF2BP3	RBM24	SFRS2IP
BCLAF1	KHDRBS1	RBM25	SFRS8
CDC5L	KHDRBS2	RBM26	SMN1
CDK12	KHDRBS3	RBM27	SNRNP70
CDK13	KHSRP	RBM28	SON
CDK7	LIN28A	RBM3	SR140
CDK9	LIN28B	RBM33	SREK1
CELF1	MBNL1	RBM34	SREK1IP1
CELF2	MBNL2	RBM38	SRP54
CELF3	MBNL3	RBM39	SRPK1
CELF4	MEX3A	RBM4	SRPK2
CELF5	MEX3B	RBM41	SRPK3
CELF6	MEX3C	RBM42	SRRM1
CLASRP	MEX3D	RBM45	SRRM2
CLK1	MSI1	RBM46	SRRM3
CLK2	NEK2	RBM47	SRRM4
CLK3	NONO	RBM4B	SRSF1
CLK4	NOVA1	RBM5	SRSF10
DAZAP1	NOVA2	RBM6	SRSF11
ELAVL1	PARP1	RBM7	SRSF12
ELAVL2	PCBP1	RBM8A	SRSF2
ELAVL3	PCBP2	RBMS1	SRSF3
ELAVL4	PCBP3	RBMS2	SRSF4
ESRP1	PCBP4	RBMS3	SRSF5
ESRP2	PNISR	RBMX	SRSF6
EWSR1	PTBP1	RBMX2	SRSF7
FMR1	PTBP2	RBMXL1	SRSF8
FUS	PUF60	RBMY1A1	SRSF9
HNRNPA0	PUM1	RBMY1B	SUGP2
HNRNPA1	PUM2	RBMY1D	SYNCRIP
HNRNPA1L2	QKI	RBMY1E	TAF15
HNRNPA2B1	RALY	RBMY1F	TARDBP
HNRNPA3	RALYL	RBMY1J	TIA1
HNRNPAB	RAVER1	RBPMS	TIAL1
HNRNPC	RAVER2	RBPMS2	TRA2A
HNRNPCL1	RBFOX1	SAFB	TRA2B
HNRNPD	RBFOX2	SAFB2	U2AF1
HNRNPDL	RBFOX3	SCAF11	U2AF2
HNRNPF	RBM10	SCAF4	U2SURP
HNRNPH1	RBM11	SCAF8	YBX1
HNRNPH2	RBM12	SF1	YBX2
HNRNPH3	RBM12B	SF3B1	YTHDC1
HNRNPK	RBM14	SF3B5	YTHDC2
HNRNPL	RBM15	SFPQ	ZC3H10
HNRNPLL	RBM15B	SFRS12	ZC3H13
HNRNPM	RBM16	SFRS12IP1	ZC3H14
HNRNPR	RBM17	SFRS14	ZC3H15
HNRNPU	RBM19	SFRS15	ZC3H8
HNRNPUL1	RBM20	SFRS16	ZRSR1
HNRNPUL2	RBM22	SFRS17A	ZRSR2

Supplementary table 3.							
List of genes regulated in MEX3A-depleted cells.							
gene	Fold change (siCTRL vs siMEX3A-sp)	p value		gene	Fold change (siCTRL vs siMEX3A-sp)	p value	
MEX3A	65,61452663	1,55277E-13		HMOX1	149,6235656	6,26405E-34	down
ALDH1A3	51,40436516	2,24842E-32		LHX1	26,79168325	2,24842E-32	up
MAP4K5	36,98281798	3,32636E-20		RP11-284F	15,73982864	9,33422E-10	
MCFD2	27,9898132	1,51781E-32		TXNIP	14,52111618	1,20956E-16	
YIF1B	26,6072506	5,35447E-08		LINC00152	13,30454418	6,98225E-16	
SYNJ2BP	26,0615355	2,19096E-15		SESN2	13,21295634	2,86055E-12	
AP1S2	23,60478233	1,82989E-12		HBEGF	10,51961874	6,81172E-06	
OTUD6B	23,33458062	4,93323E-15		GADD45A	10,25651926	1,72523E-13	
TAF2	22,69864852	7,39078E-14		BLOC1S2	9,81747943	8,75552E-09	
NFIB	22,49054606	1,34458E-12		RNF24	9,462371614	6,21129E-06	
USP12	22,23309891	3,61193E-16		FHOD1	9,036494737	3,80032E-06	
TMEM30A	21,18361135	3,25967E-16		BLVRB	8,912509381	4,74134E-09	
STX2	21,0377844	6,8375E-11		SAT1	8,912509381	1,40371E-11	
PLAU	21,0377844	3,4107E-06		EGR1	8,729713684	2,33236E-12	
ZNF511	20,65380156	5,33152E-12		CAMK2G	8,550667129	1,48657E-09	
ATP6V1A	20,46444637	2,57668E-16		ZFP36L1	8,356030182	1,0762E-09	
TAF1B	20,18366364	3,46965E-08		FAM222B	8,14704284	4,65806E-08	
CCDC68	18,7068214	9,28765E-20		TBC1D13	7,691304403	1,26483E-06	
ZNF618	18,62087137	0,000302043		AP5Z1	7,691304403	7,42973E-05	
ANTXR2	18,28100216	2,19096E-15		CAMTA2	7,413102413	0,00053025	
PSMD6	17,25837892	2,90688E-09		SERPINB9	7,261059574	9,84388E-06	
PPP2CB	16,82674061	1,37987E-14		MIR4435-2	7,261059574	2,45278E-09	
ARPC1A	16,67247213	7,68918E-17		QPRT	7,227698036	3,38992E-07	
ARSK	15,6675107	1,07589E-10		PPP1R15A	6,902398038	1,28426E-06	
GLO1	15,31087462	1,42979E-16		ASNS	6,760829754	0,000113878	
ALDH9A1	14,19057522	9,22103E-15		SLC1A5	6,714288529	0,003551417	
BTBD10	13,89952631	4,12739E-09		SQSTM1	6,280583588	7,13158E-05	
TXNDC12	13,39676687	3,13014E-14		JOSD2	5,701642723	0,011821907	
TMED5	13,30454418	2,501E-12		PSMC4	5,61047976	0,00020949	
GPBP1L1	12,76438809	1,83877E-12		PDLIM4	5,597576015	0,001680109	
FAM73A	12,41652308	5,63958E-09		ZNF346	5,584701947	0,003286808	
TDG	12,35947433	2,96109E-05		UBC	5,395106225	1,10933E-08	
XRCC6BP1	12,0503594	2,30601E-05		ANKRD52	5,357966575	0,00176051	
ZNF146	11,24604974	0,000102116		DPCD	5,308844442	3,75283E-05	
BROX	10,93956366	1,56648E-06		CBR3	5,284452518	7,86554E-05	
WDR36	10,88930093	2,54533E-08		RP11-445F	5,272298614	0,001506442	
PRNP	10,71519305	1,39498E-12		ZNFX1	5,069907083	1,90307E-05	
MAP2K4	10,61695557	5,69056E-07		GLRX2	5,035006088	0,00054515	
PLAC8	10,54386896	2,10709E-05		LGALS8	4,954501908	0,000130181	
RNF182	10,51961874	1,195E-12		KATNB1	4,886523593	0,001106502	
EDEM3	10,32761406	3,38992E-07		MAT2A	4,864072057	0,020491716	
NAB1	10,23292992	3,50407E-07		ATP6V0A1	4,841723676	0,000103652	
DHX33	10,0461579	5,82119E-10		KLHL21	4,764309868	0,000456076	
SPAST	9,727472238	4,30733E-06		RAB11FIP5	4,742419853	0,0124503	

DDX21	9,638290236	3,52001E-22		R3HDM4	4,742419853	0,000141614	
AFAP1	9,616122784	7,86494E-08		PSMD14	4,709773264	0,000838916	
FBXW11	9,571940713	1,04092E-05		TOLLIP	4,698941086	0,000526309	
CSDE1	9,54992586	2,09273E-21		MED31	4,666593803	4,80528E-05	
MYBL1	9,462371614	4,91599E-11		ATG2A	4,591980128	0,001582662	
TERC	9,397233106	5,26594E-08		LMLN	4,591980128	0,000260272	
RP11-582E3.6	9,397233106	1,15865E-05		SLC2A3	4,518559444	0,00018442	
ADCK2	9,375620069	6,21129E-06		NFKBIB	4,466835922	0,01736279	
CISD2	8,953647655	1,83269E-06		MAFG	4,436086439	0,002929016	
PDHX	8,590135215	1,57126E-09		OSER1	4,415704474	0,010177388	
TULP3	8,433347578	3,46965E-08		PFN2	4,405548635	1,26897E-05	
IPO8	8,433347578	1,3936E-11		EMC3	4,385306978	7,59149E-05	
SRGAP2C	8,222426499	7,59149E-05		SLC3A2	4,385306978	9,18989E-05	
C5orf24	8,109610579	1,521E-07		DOT1L	4,305266105	0,002664272	
STXBP5	8,053784412	4,92336E-05		TRIAP1	4,295364268	0,003393131	
YWHAZ	7,925013305	5,32152E-10		STK40	4,275628862	0,000208276	
AEBP2	7,90678628	2,25622E-05		PPP2R1B	4,265795188	0,00021539	
UGCG	7,870457897	6,68225E-07		ZRSR2	4,226686143	0,001194195	
PRKAR2B	7,852356346	3,69101E-06		KLHDC10	4,216965034	0,001739538	
RIN2	7,6207901	6,40205E-07		CD276	4,187935651	0,001506372	
BLOC1S6	7,603262769	7,27126E-08		SNHG19	4,159106105	0,001358331	
BIRC2	7,498942093	1,41509E-11		RHBDD2	4,120975191	0,003106456	
GPX8	7,430191379	1,89522E-05		MKNK2	4,111497211	6,46834E-07	
TMED2	7,413102413	8,3423E-08		MKKS	4,111497211	0,000996585	
LLPH	7,396052751	1,521E-07		EVI5L	4,111497211	0,001174305	
GBAS	7,379042301	2,49173E-06		HMGCS1	4,092606597	0,004722734	
PPFIBP2	7,345138682	0,000768801		SWI5	4,073802778	0,025831715	
VANGL1	7,244359601	0,000173529		AK5	4,055085354	0,001855626	
DENND5B	7,227698036	5,20735E-10		TALDO1	4,055085354	0,000421005	
NDUFV2	7,144963261	9,95251E-12		BAG3	4,008667176	0,000616441	
GSTCD	7,128530301	0,011661471		CLIP4	3,999447498	0,009565814	
PSMA3-AS1	7,128530301	0,000476643		STX1A	3,990249024	0,016002398	
MIB1	7,09577768	1,36959E-06		WBP2	3,990249024	0,026907381	
PDCD4	7,04693069	2,9771E-05		TFE3	3,917418771	4,9215E-05	
HELLS	6,902398038	1,02725E-08		HTATIP2	3,917418771	0,001607799	
TRRAP	6,886522963	6,18834E-05		TTC1	3,917418771	3,34526E-06	
SLC30A7	6,854882265	1,42645E-06		CCDC174	3,908408958	0,000583854	
RBPJ	6,839116473	1,26551E-06		DCP1A	3,899419867	0,010104591	
SECISBP2	6,745280277	2,45143E-06		PLAUR	3,88150366	0,000673058	
PPIP5K2	6,729766563	8,28281E-08		SS18L2	3,819442708	0,0320913	
NFKB1	6,714288529	0,002742588		ANKRA2	3,801893963	0,008620287	
DAB2IP	6,668067692	0,000130181		DUSP14	3,801893963	0,000799697	
BAG5	6,622165037	4,29499E-07		ATP6V0E1	3,784425847	5,98248E-05	
AGL	6,60693448	0,000579895		SLC7A5	3,775721909	8,21203E-05	
CTDSPL2	6,546361741	1,64299E-07		DAGLB	3,715352291	0,046621873	
ME2	6,516283941	6,59372E-06		AEN	3,715352291	0,019995136	
SSX2IP	6,441692655	7,45382E-06		PPP1R26-A	3,706807218	0,022686657	
NAA20	6,412095766	0,009983713		SNHG9	3,706807218	0,000878544	
DNAJC16	6,367955209	4,9215E-05		FBXO30	3,672823005	0,005632315	
HABP4	6,338697113	2,99573E-05		MPHOSPH	3,655947916	0,000774055	

HIST1H2BF	6,338697113	0,013542537		ZNF367	3,630780548	0,010341364	
PSMD10	6,324118514	0,000104525		MINK1	3,580964371	0,032526281	
EMP1	6,309573445	1,28441E-11		AIFM2	3,572728382	0,000329781	
SLC5A3	6,295061829	1,64499E-08		ZFAND5	3,564511334	0,002692843	
RRN3	6,280583588	0,000151302		DVL3	3,539973411	0,006306374	
HIST1H1D	6,266138647	0,00074207		C6orf1	3,539973411	0,015096852	
AK4	6,251726928	2,67979E-09		GRAMD3	3,531831698	0,000248707	
C17orf58	6,251726928	0,001040075		SYNJ1	3,531831698	0,004939027	
CCM2	6,165950019	3,79872E-06		TOMM34	3,52370871	0,000130181	
MAP3K13	6,151768727	7,39871E-06		NMRAL1	3,515604405	0,033285988	
BRI3BP	6,137620052	4,05336E-07		WEE1	3,515604405	0,01210205	
PNMA2	6,081350013	0,0003438		CEP170B	3,475361614	0,027193887	
LSM14B	6,011737375	0,0002555		TMEM59	3,475361614	0,018097687	
TRIM25	5,970352866	3,51743E-07		PDZD11	3,475361614	0,00015746	
TCF7	5,929253246	1,67333E-09		MAX	3,443499308	0,046890311	
ORMDL1	5,929253246	4,85428E-06		SIRT7	3,427677865	0,004450163	
POLR3K	5,821032178	0,001052175		EIF3J	3,396252726	4,91825E-06	
NOL9	5,807644175	2,0481E-05		PUS3	3,396252726	0,026151189	
TAF8	5,794286964	5,84644E-05		FBXW7	3,388441561	0,001201615	
VMA21	5,780960474	3,42801E-06		PSMD13	3,380648362	0,001358331	
TM4SF18	5,780960474	3,2199E-06		LIMK1	3,3419504	0,032512552	
RBM41	5,754399373	0,001896441		KIAA1143	3,3419504	0,013452269	
MRGBP	5,72796031	2,27092E-06		WIZ	3,318944576	0,012284543	
PPP2R5A	5,714786367	3,52961E-05		SERPINB8	3,296097122	0,021065915	
CD46	5,688529308	7,92319E-06		TTLL4	3,265878322	0,033636494	
MAD2L1	5,597576015	0,004390802		ERI3	3,221068791	0,045661576	
SUCO	5,571857489	0,000309568		BTG1	3,221068791	0,013312245	
LAMP2	5,508076964	7,86554E-05		TRIM11	3,221068791	0,007337241	
JADE1	5,495408739	6,9828E-06		PSMD11	3,191537855	0,032930546	
GNPTAB	5,495408739	0,000700095		CSK	3,184197522	0,038705509	
KATNBL1	5,432503315	0,002692843		CCDC34	3,184197522	0,01131695	
ATP2B4	5,420008904	1,10933E-08		ABCC5	3,16227766	0,045757422	
ECT2	5,420008904	1,28098E-05		UBXN4	3,16227766	0,000878544	
CAMKK2	5,407543229	0,001757264		RGS3	3,155004623	0,012390112	
FAIM	5,407543229	0,007533654		NSFL1C	3,140508694	0,000476643	
ESCO2	5,370317964	0,003318564		JOSD1	3,133285724	0,009184612	
SENP5	5,357966575	0,001082528		TMEM208	3,133285724	0,004170785	
STRN	5,321082593	0,000103785		NT5C2	3,111716337	0,015179561	
FAM199X	5,321082593	0,001156594		TMEM170/	3,111716337	0,013680938	
EPT1	5,321082593	0,001506648		FAM98A	3,104559588	0,003286808	
IPO7	5,321082593	1,0989E-08		HAUS8	3,104559588	0,012538375	
SPCS3	5,272298614	0,000732874		NPLOC4	3,104559588	0,000798884	
RHOT1	5,260172664	0,007284705		TSR3	3,054921113	0,010004727	
NCOA3	5,248074602	0,000943677		BCAP31	3,054921113	0,037944869	
UBA6	5,22396189	0,000279307		PSMD3	3,040885026	0,022951973	
YES1	5,211947111	3,60452E-09		DAP3	3,040885026	0,000568237	
UBE2E3	5,188000389	8,66322E-06		ATP6V1B2	3,033891184	0,047744857	
SLC39A10	5,17606832	2,49173E-06		SH3BGRL3	3,026913428	0,003560253	
CALU	5,164163693	1,26551E-06		RNF181	3,026913428	0,014766728	
ELOVL5	5,140436516	6,74847E-05		BAK1	3,006076303	0,014124291	

HIPK1	5,140436516	0,001855626		CLU	3,006076303	0,000747794	
CCDC43	5,140436516	2,49173E-06		AKIRIN2	2,985382619	0,046710726	
AGPAT3	5,116818355	8,50321E-05		TRIM38	2,951209227	0,031645786	
FAM208A	5,081594426	0,000107671		KLC1	2,937649652	0,041901934	
ZNF714	5,046612976	0,003963984		C9orf142	2,910717118	0,013250016	
PAQR4	5,035006088	0,007337241		PSAP	2,877398415	0,003539324	
CDC42SE1	5,00034535	0,01959414		GRIPAP1	2,870780582	0,00340261	
SEH1L	4,97737085	0,015811054		PGD	2,870780582	0,023595852	
SMCHD1	4,94310687	2,30667E-05		ARF4	2,857590543	0,030596929	
ITGB4	4,94310687	3,18279E-07		STX4	2,844461107	0,01260166	
C2orf69	4,93173804	6,81373E-07		UBE2O	2,837919028	0,016064306	
CMTM7	4,920395357	2,49976E-06		TAF1C	2,805433638	0,030865523	
R3HDM2	4,920395357	0,001095537		CHORDC1	2,805433638	0,009207028	
YAP1	4,886523593	0,001651322		CBX6	2,79898132	0,017289185	
LMO4	4,864072057	0,012115984		BLMH	2,786121169	0,017027481	
RPL41	4,864072057	0,032634525		ATP6V0D1	2,786121169	0,013267123	
HEXIM1	4,83058802	3,50554E-06		ATP5L	2,786121169	0,028384976	
TBL1XR1	4,797334486	6,85661E-05		ZNF579	2,773320105	0,032664599	
ENTPD4	4,73151259	0,000514452		GSTP1	2,766941645	0,030482755	
RC3H2	4,720630413	6,08577E-05		KIAA1715	2,766941645	0,033253695	
HMGN2	4,698941086	0,000208276		IGF2BP3	2,760577856	0,002079115	
BLM	4,677351413	0,010350611		DHCR24	2,747894153	0,012658838	
CFD	4,677351413	0,001384779		PPP1R18	2,741574172	0,010760314	
FEM1A	4,666593803	6,00064E-05		CAMLG	2,741574172	0,000921511	
ADAM10	4,645152752	4,80528E-05		RNF10	2,716439269	0,030322133	
TEAD2	4,634469197	0,028682079		NDST1	2,716439269	0,024365397	
PHACTR2	4,613175746	0,004722734		SELK	2,716439269	0,017027481	
FAM35A	4,613175746	0,003215856		GSTO1	2,716439269	0,014747918	
DTL	4,602565736	5,08571E-05		TNFRSF1A	2,703958364	0,000414219	
CDCA7	4,591980128	0,001954423		IER3IP1	2,703958364	0,030297045	
EXTL2	4,570881896	0,001229783		GNPDA1	2,691534804	0,005165533	
CYP27C1	4,539416167	0,000798884		PLPP1	2,685344446	0,022751581	
FBXO27	4,518559444	0,007261139		STK17A	2,685344446	0,006695908	
ZNF580	4,508167045	0,030865523		PSMB1	2,673006409	0,033636494	
LINC00657	4,477133042	9,19848E-06		PSME4	2,673006409	0,007473006	
RAB22A	4,466835922	7,63925E-05		PSMB6	2,673006409	0,001936365	
SEPT10	4,456562484	5,59445E-05		USP14	2,654605562	0,000700095	
CAV2	4,436086439	6,46834E-07		DNTTIP1	2,630267992	0,030677975	
CREBL2	4,436086439	6,31595E-05		USP36	2,624218543	0,022631579	
EXOC5	4,425883724	0,006535215		SHC1	2,624218543	0,002633283	
MZT1	4,425883724	0,00206274		ACAT2	2,618183008	0,022751581	
SLC35F2	4,375221052	0,000273894		TCEA2	2,618183008	0,045931743	
RREB1	4,375221052	0,004494523		MED15	2,600159563	0,019839624	
KIF3A	4,375221052	0,001019553		HMGA1	2,600159563	0,004583565	
MAT2B	4,355118737	0,00910443		ELP5	2,594179362	0,0320913	
TMPO	4,305266105	5,69056E-07		TWF2	2,594179362	0,023506929	
SPIN1	4,295364268	0,00075023		KPNA1	2,588212915	0,024336804	
RNF149	4,295364268	0,004427804		LASP1	2,576321157	0,027320925	
FAM168B	4,285485204	0,001905688		UBR4	2,576321157	0,036396688	
CCDC112	4,265795188	0,007014009		UFD1L	2,570395783	0,003281905	

PIAS2	4,255984131	0,021270645		SMIM4	2,564484037	0,045246961	
OGFRL1	4,255984131	0,001040075		EIF1	2,558585887	0,001916414	
CLMN	4,255984131	0,039768522		MSN	2,552701303	0,004007727	
OSBPL8	4,23642966	2,75769E-05		JUN	2,552701303	0,001953072	
APOO	4,23642966	0,023426654		SPATS2	2,546830253	0,048176744	
SMC2	4,226686143	8,3601E-05		UBALD2	2,540972706	0,020699066	
HIST1H1C	4,226686143	0,026515095		TAX1BP1	2,511886432	0,007124928	
SAP30	4,216965034	0,002143333		FHL2	2,488857318	0,012115984	
KRT80	4,216965034	0,000476843		RGS4	2,477422058	0,008483676	
SAPCD2	4,187935651	0,000108917		POMP	2,460367604	0,033282756	
PUDP	4,168693835	0,011552585		SLC25A37	2,454708916	0,019655623	
CLOCK	4,168693835	0,004057027		CCDC186	2,437810818	0,009618	
DCBLD1	4,159106105	0,005306502		SLC25A13	2,432204009	0,024791577	
FGFR1OP2	4,139996748	8,71441E-05		LYPLA2	2,421029047	0,047930176	
PFAS	4,13047502	0,006664023		UBE2A	2,409905429	0,046474756	
AAMP	4,120975191	0,001651322		AHSA1	2,409905429	0,019331332	
FIGNL1	4,120975191	0,010278819		NDUFA5	2,393315756	0,030482755	
SLC38A1	4,111497211	0,00010989		LAPTM4A	2,382319469	0,027502498	
NLN	4,10204103	0,00035911		PRDX5	2,376840287	0,02640027	
POGK	4,092606597	0,000475725		KMT2C	2,355049284	0,03626595	
GNE	4,073802778	0,006002743		PSMA2	2,344228815	0,032664599	
KLHL8	4,064433292	0,000217506		WDR83OS	2,338837239	0,019584962	
AKAP12	4,027170343	0,003318564		PPIF	2,338837239	0,04390072	
BOD1	4,027170343	0,002376355		ERCC1	2,31739465	0,017903621	
SERTAD2	4,017908108	0,044603358		STK4	2,31206479	0,016574635	
PTPRA	3,999447498	0,024663112		KIF3B	2,31206479	0,036945648	
MPC1	3,990249024	0,01141245		GPAT3	2,31206479	0,01498325	
LNPEP	3,990249024	0,011819278		DYNC1H1	2,306747189	0,045246961	
TGFBR3	3,971915495	0,004939027		PAIP2	2,285598803	0,039357076	
GSPT2	3,971915495	0,005118519		POLE3	2,275097431	0,022751581	
ABHD10	3,962780343	0,000255662		MAP3K3	2,269864852	0,035624881	
LIMA1	3,953666201	1,52254E-05		MCL1	2,264644308	0,028218989	
APIP	3,944573021	0,014263845		HGS	2,264644308	0,043233468	
CKB	3,935500755	0,022303243		SAMD4B	2,202926463	0,025831715	
ANKRD50	3,926449354	0,000283771		YWHAG	2,197859873	0,010375599	
CNOT3	3,917418771	0,027016387		KCMF1	2,192804935	0,021336327	
PHF6	3,908408958	3,40181E-05		ATOX1	2,182729912	0,027502498	
SEMA3C	3,899419867	0,000611853		LRP10	2,162718524	0,017477911	
ID2	3,89045145	0,00074207		PSMD4	2,157744409	0,042252616	
MAPK1IP1L	3,89045145	0,000283771		PSMB2	2,118361135	0,016587407	
SLC12A2	3,837072455	0,000617858		KRT19	2,11348904	0,047930176	
PRKD3	3,828247433	0,001953913		MAF1	2,11348904	0,022108766	
INIP	3,828247433	0,003103997		PPP2CA	2,10862815	0,022108766	
ERLIN2	3,810658234	0,004950788		PSMA7	2,084490883	0,044671032	
HINT3	3,79314985	0,019040726		PCYT1A	2,065380156	0,029324802	
EIF4H	3,775721909	0,007124928		TKT	2,055890596	0,04346265	
MFI2	3,775721909	0,019992654		PSMD2	2,018366364	0,018488402	
DCP2	3,775721909	0,003591241		SRPK2	2,004472027	0,047744857	
HCFC1R1	3,749730022	0,001896441					
PRR11	3,741105883	1,36959E-06					

MPHOSPH9	3,723917063	0,004781289					
CDC27	3,706807218	0,000334963					
PANK3	3,672823005	0,000480646					
STARD7	3,664375746	0,000210564					
FAM101B	3,655947916	0,000100946					
MRPL16	3,647539469	0,000371201					
SPIRE1	3,630780548	0,001229783					
PDP2	3,630780548	0,000636953					
DEPDC1	3,622429984	0,012928711					
KCTD3	3,622429984	0,014124291					
PBRM1	3,589219346	0,001284167					
GK5	3,589219346	0,006382678					
ZMYM4	3,572728382	0,000421005					
HCG11	3,556313186	0,045246961					
METAP1	3,548133892	0,001506648					
TBX2	3,539973411	0,007570615					
DCAF10	3,515604405	2,33626E-05					
NT5E	3,515604405	2,05374E-05					
METTL13	3,49945167	0,000778611					
NEDD1	3,49945167	0,012284543					
PER3	3,475361614	0,047520703					
SFXN1	3,467368505	4,4356E-05					
SCAF11	3,459393778	9,85767E-05					
MYADM	3,435579479	3,47671E-05					
FKBP3	3,427677865	0,000422543					
FUBP3	3,411929116	0,000760339					
TMEM181	3,404081897	0,008258997					
BTN2A1	3,396252726	0,016587407					
FAM213A	3,372873087	0,016247991					
EREG	3,372873087	0,001311511					
CLPX	3,365115694	0,012115984					
SBNO1	3,357376142	4,36465E-05					
MRPL48	3,357376142	0,004497354					
MGAT5	3,349654392	0,034417351					
ING2	3,349654392	0,010627931					
UBXN2A	3,334264128	0,005586382					
ARHGEF12	3,318944576	0,005115671					
CREB1	3,311311215	0,003591241					
HS2ST1	3,311311215	0,038944335					
PTPN1	3,311311215	0,000110656					
NUP58	3,288516309	0,000273894					
FAM20B	3,280952931	0,015577459					
SWAP70	3,280952931	0,000557915					
SAMD1	3,265878322	0,002978457					
ETV6	3,25836701	0,016247991					
PPM1B	3,250872974	0,036288323					
LRRFIP1	3,235936569	7,7924E-05					
FGFR1OP	3,235936569	0,018720136					
EIF4A1	3,221068791	5,94701E-05					
TOR1AIP2	3,206269325	5,57222E-05					

RBL1	3,19889511	0,01912525					
PNN	3,19889511	0,003439524					
IARS2	3,191537855	0,001388932					
UBE3B	3,184197522	0,032930546					
RSBN1	3,16227766	0,018097687					
RBM28	3,16227766	0,000182927					
FBXO33	3,155004623	0,028795461					
KLHL23	3,155004623	0,010375599					
CHD5	3,147748314	0,011199823					
BRWD1	3,147748314	0,011552585					
PHF20L1	3,133285724	0,000985222					
KEAP1	3,118889584	0,030596929					
FURIN	3,111716337	0,047960665					
TNRC6B	3,104559588	0,000450782					
PTBP3	3,104559588	0,001229783					
PDCL3	3,097419299	0,017679091					
PHF10	3,097419299	0,016587407					
MIEN1	3,097419299	0,00705872					
HNRNPLL	3,08318795	0,040518669					
EI24	3,08318795	0,006241912					
RRM2	3,08318795	0,001229783					
ATP1B1	3,069021988	0,004722734					
CSNK1G3	3,069021988	0,019168384					
VPS36	3,061963434	0,015577459					
TMEM214	3,054921113	0,043173316					
FAR1	3,04789499	0,013095356					
FERMT1	3,040885026	0,003014119					
KIF14	3,040885026	0,00804679					
REEP5	3,033891184	0,006953093					
HNRNPR	3,026913428	0,001506648					
C11orf24	3,026913428	0,033954105					
OSBPL3	3,01995172	0,013726027					
ERCC3	3,01995172	0,039768522					
ZBTB18	3,01995172	0,013150844					
MT1A	3,01995172	0,038982563					
PEG10	3,013006024	0,020551162					
AMOTL2	3,006076303	0,004095028					
NCAPH	2,999162519	0,01498325					
WWC2	2,999162519	0,001885786					
SNHG12	2,992264637	0,027823085					
KAT6B	2,985382619	0,012904838					
LRRC58	2,985382619	9,44891E-05					
SMC1A	2,978516429	0,010235547					
PDCD2	2,971666032	0,006109367					
CCDC50	2,971666032	0,001910821					
MAGT1	2,96483139	0,030322133					
HDAC8	2,96483139	0,010054814					
MRPL19	2,951209227	0,047030571					
ADNP	2,944421634	0,022384224					
QKI	2,944421634	0,00021539					

ARHGAP12	2,944421634	0,03604242						
CAND1	2,937649652	0,004915575						
TYMS	2,937649652	0,000811396						
ZNF638	2,924152378	0,007337241						
SPAG5	2,917427014	0,01141245						
RAP2B	2,917427014	0,009176136						
PTGR1	2,910717118	0,038561386						
COQ10B	2,910717118	0,041901934						
THOC7	2,910717118	0,01078239						
HIP1	2,890679882	0,006671327						
TIPRL	2,890679882	0,010684814						
BICD2	2,890679882	0,011083412						
MCM10	2,870780582	0,00130732						
SNX13	2,857590543	0,021989086						
GJC1	2,857590543	0,008995433						
USP13	2,851018268	0,003878459						
RAB17	2,851018268	0,033782865						
UQCC2	2,851018268	0,017244956						
GCLM	2,844461107	0,001201615						
MED1	2,844461107	0,000195961						
G2E3	2,824879975	0,026443518						
TNNT1	2,824879975	0,023595852						
PPP1R12C	2,818382931	0,028795461						
VARS2	2,818382931	0,033686139						
SLC39A6	2,81190083	0,014151282						
CRIM1	2,805433638	0,021082071						
ZC3H14	2,792543841	0,030482755						
CENPH	2,792543841	0,047195229						
FUT11	2,779713268	0,02960336						
RAD17	2,773320105	0,049243383						
SP3	2,773320105	0,047744857						
MBTPS2	2,766941645	0,012981844						
CELF1	2,766941645	0,0001573						
FKBP14	2,760577856	0,023861576						
CALR	2,760577856	0,001793795						
FAM107B	2,754228703	0,038944335						
ROCK1	2,754228703	0,001409248						
WDR12	2,754228703	0,008751097						
MRE11A	2,747894153	0,024501598						
DGKD	2,747894153	0,01896888						
PCNX	2,747894153	0,037344055						
MYH10	2,722701308	0,005951139						
UGP2	2,710191632	0,01959414						
CYP20A1	2,703958364	0,00917966						
DGKE	2,703958364	0,030141878						
CBX1	2,697739432	0,008906323						
TBRG4	2,697739432	0,005917393						
PMEPA1	2,691534804	0,008420826						
AKAP1	2,685344446	0,02864504						
TGOLN2	2,666858665	0,000799697						

RAB8A	2,66072506	0,003177266					
ERLEC1	2,648500139	0,04346265					
DVL1	2,648500139	0,013175229					
RNF20	2,648500139	0,013550784					
SNRNP25	2,642408757	0,035012183					
ACSL3	2,636331386	0,004583565					
DEK	2,630267992	0,005274827					
NOM1	2,588212915	0,007451789					
ZBTB38	2,576321157	0,008432684					
MYO18A	2,570395783	0,03604242					
CAMSAP2	2,564484037	0,008818754					
FLII	2,564484037	0,047897761					
UBLCP1	2,552701303	0,039212605					
EPB41	2,546830253	0,020027338					
HECTD1	2,540972706	0,023506929					
CASC3	2,540972706	0,008765047					
HSPA4L	2,540972706	0,025983641					
GTF3C6	2,529297996	0,018585766					
RPL5	2,517676928	0,025026241					
GOLIM4	2,517676928	0,007930925					
SMAD2	2,506109253	0,020224648					
FNIP1	2,506109253	0,045246961					
PDGFC	2,500345362	0,020216069					
BDP1	2,488857318	0,023625258					
DNAJC10	2,483133105	0,026685928					
SERBP1	2,483133105	0,004439282					
GOPC	2,477422058	0,039407745					
MMS22L	2,477422058	0,033059192					
C6orf106	2,477422058	0,011024102					
TIMM17A	2,471724145	0,014860284					
PDIA4	2,471724145	0,006902242					
PRKCI	2,471724145	0,037534902					
CSNK2A2	2,460367604	0,013328679					
EIF4G1	2,454708916	0,003281905					
TOMM40	2,449063242	0,034691695					
ZC3H11A	2,443430553	0,033954105					
MAP3K2	2,437810818	0,01498325					
COG8	2,437810818	0,026257408					
SIX1	2,421029047	0,032540685					
DDX17	2,415460834	0,016189942					
UBTF	2,409905429	0,030141878					
ZMYND11	2,4043628	0,006671327					
ID1	2,4043628	0,042752165					
LARS	2,393315756	0,011960636					
TJP1	2,387811283	0,016713988					
GMEB1	2,382319469	0,018461684					
SGOL2	2,371373706	0,03477749					
USO1	2,365919697	0,04346265					
SRSF11	2,355049284	0,037993712					
PDCD10	2,349632821	0,034071863					

IBTK	2,344228815	0,027573017					
IFITM2	2,344228815	0,019040726					
SF3A1	2,333458062	0,043008026					
ACTR3	2,322736796	0,014689075					
CALD1	2,322736796	0,017027481					
SEMA3A	2,31739465	0,032403943					
PHF19	2,306747189	0,015274555					
DARS	2,296148648	0,005499248					
CKAP4	2,280342072	0,030778455					
UBXN7	2,280342072	0,038032891					
CEP78	2,275097431	0,046687125					
ADI1	2,275097431	0,020842704					
TTL	2,269864852	0,04346265					
NR3C1	2,249054606	0,02940522					
PPT1	2,249054606	0,028097012					
MAP7D3	2,243881924	0,025486146					
CDC42EP3	2,233572223	0,022987017					
PRKAR1A	2,228435149	0,007337241					
CDK6	2,223309891	0,008682141					
DYNC1LI2	2,208004733	0,00894857					
NUCB2	2,202926463	0,0320913					
MRPS16	2,202926463	0,040989811					
MFHAS1	2,187761624	0,028787417					
DR1	2,182729912	0,049737833					
IFITM3	2,177709772	0,028489354					
SMARCB1	2,172701179	0,038467825					
CCDC88A	2,167704105	0,04260296					
TSN	2,167704105	0,029504922					
CIAPIN1	2,152781735	0,030865523					
KNSTRN	2,152781735	0,047930176					
PTBP1	2,118361135	0,020144146					
MRPS18B	2,098939884	0,024020433					
PUM3	2,084490883	0,020318947					
RAD21	2,079696687	0,012551082					
SSU72	2,074913517	0,034153857					
HMGB2	2,065380156	0,034321429					
PDLIM2	2,046444637	0,026907381					
BCLAF1	2,041737945	0,049531316					
HNRNPC	2,023019179	0,049695223					
UBAC1	2,018366364	0,048762481					
MT2A	2,009092813	0,02099408					
